# Gender and disciplinary differences in grant proposal peer review: Content and sentiment in 39,280 reports

**DOI:** 10.1371/journal.pone.0352900

**Published:** 2026-09-15

**Authors:** Stefan Müller, Gabriel Okasa, Michaela Strinzel, Anne Jorstad, Katrin Milzow, Matthias Egger

**Affiliations:** 1 School of Politics and International Relations, University College Dublin, Dublin, Ireland; 2 Swiss National Science Foundation, Bern, Switzerland; 3 Institute of Social and Preventive Medicine, University of Bern, Bern, Switzerland; 4 Center for Reproducible Science and Research Synthesis, University of Zurich, Zurich, Switzerland; 5 Population Health Sciences, Bristol Medical School, University of Bristol, Bristol, United Kingdom; Xi’an Jiaotong University, CHINA

## Abstract

Peer review by experts is central to the evaluation of grant proposals, but little is known about how gender and disciplinary differences shape the content and tone of grant peer review reports. We analyzed 39,280 review reports submitted to the Swiss National Science Foundation between 2016 and 2023, covering 11,385 proposals for Project Funding across 21 disciplines from the Social Sciences and Humanities (SSH), Life Sciences (LS), and Mathematics, Informatics, Natural Sciences, and Technology (MINT). Using supervised machine learning, we classified over 1.3 million sentences by evaluation criteria and sentiment. Reviews in SSH were significantly longer and more critical, with less focus on the applicant’s track record, while those in MINT were more concise and positive, with a greater focus on the track record, as compared to those in LS. Compared to male reviewers, female reviewers wrote longer reviews that more closely aligned with the evaluation criteria and expressed more positive sentiments. Female applicants tended to receive reviews with slightly more positive sentiment than male applicants. The tone, length, and focus of peer review reports varied systematically by applicant gender and disciplinary context. These differences have important implications for fairness and consistency in research funding.

## Introduction

Peer review is central to the allocation of research funding, aiming to ensure that resources are distributed fairly on the basis of merit, scientific quality, and potential. Typically, experienced external reviewers provide structured assessments of proposals that inform the recommendations made by evaluation panels. The process of grant peer review has, however, been criticized for many years [1]. Reviewers often remain anonymous, applicants may not see full review reports, and the weight given to the external reports in funding decisions is generally unclear [2–5].

Compared with journal article review, peer review of research grant proposals focuses on the feasibility of a proposed project [6] and requires reviewers to forecast likely outcomes rather than evaluate completed work [7]. Grant review also typically follows a single-round process with no opportunity for revision, whereas journal peer review usually involves one or more rounds of revision and resubmission. Recent evidence shows that researchers report lower trust in grant review than in manuscript review [8]. Moreover, peer review of both articles and grant proposals may be influenced by the gender and other characteristics of applicants and reviewers, potentially introducing bias [9–13]. Approaches to peer review may vary across research areas or disciplines. Disciplinary differences and cultures shape how proposals are reviewed, with different fields emphasizing the applicants’ track record, methodological rigor, or originality. The well-documented low inter-rater reliability in peer review underscores the difficulty of achieving consistent evaluations across experts [14–16].

Although billions of research dollars are distributed competitively using peer review, the process remains under-researched. While journal peer review and editorial practices have been the focus of a growing body of empirical research [17–22], the evidence on grant peer review is scarce [23]. Wessely has argued that studying grant peer review may be more important than studying publication practices: whereas journal articles reflect completed research, rejected grant proposals often represent studies that will never be done [24].

Funding success strongly shapes academic careers. At the Netherlands Organization for Scientific Research, applicants just above the funding threshold later secured more than twice as much funding as those just below [25], a finding replicated across 14 programs from six funders [26]. Grant review decisions thus have lasting effects on research and careers [27], making it essential to study the process for fairness and transparency.

The Swiss National Science Foundation (SNSF) supports basic research and use-inspired basic research across all disciplines. Its largest funding scheme, Project Funding, supports investigators pursuing self-chosen topics. Project proposals are reviewed by at least two external experts, who evaluate the applicant’s track record, the research’s relevance and originality, the suitability and feasibility of the methods, and assign an overall score. We compared content and sentiment of peer review reports used to evaluate SNSF project grant proposals.

## Materials and methods

The full sample of peer review reports contained 44,026 reports written in English, German, French, or Italian, pertaining to 11,981 proposals submitted between October 2016 and April 2023 across 21 disciplines in the Social Sciences and Humanities (SSH), Life Sciences (LS), and Mathematics, Informatics, Natural Sciences, and Technology (MINT). Although reviewers in some SSH disciplines could submit their reports in these languages, most reviews are in English (see S2 Text and S1 Fig in S1 File). We restricted our analysis to English-language reviews using Google’s Compact Language Detector 2 (CLD2) [28]. This model builds on a computationally efficient Naïve Bayes classifier. Because CLD2 was optimized for texts of at least 200 characters (approximately two sentences), we applied language detection to the full text of each reviewer’s response to an evaluation criterion rather than at the sentence level. We then retained only sentences from texts classified as English. To empirically validate this approach, we manually reviewed 1,000 randomly sampled sentences: 99.1% of sentences were assigned to the correct language. The nine misclassifications comprised two cases involving literature references in other languages, one genuine language detection error, and six instances of special characters due to formatting issues. The validation demonstrates the accuracy of the approach, confirming previous studies [29].

We further excluded the few proposals with an invalid date of birth or missing gender of the applicant. Gender is self-reported, and during the period of analysis, the SNSF recorded only two categories for gender. The final analysis dataset included 39,280 English peer review reports (89.2% of total), corresponding to 11,385 proposals, with about 3.5 reviews per proposal. These reports comprised 1,304,621 sentences and 30,477,479 words.

### Characteristics of peer reviewers and applicants

Table 1 presents the distribution of review reports by research domain and reviewer and applicant characteristics. The MINT and LS domains accounted for around 15,000 reviews each, while the SSH had fewer (just under 9,000), in line with the larger number of proposals submitted in MINT and LS. Across all fields of science, there were fewer female reviewers than male reviewers. This was particularly pronounced in MINT (12.2% female reviewers), followed by LS (23.2%), with the highest contribution of female reviewers in SSH (38.5%).

**Table 1:** Distribution of review reports across research domains and reviewer and applicant characteristics. *Notes*: UAS/UTE: University of Applied Sciences or University of Teacher Education; Other professorships include honorary, titular and visiting professors; LS: Life Sciences; MINT: Mathematics, Informatics, Natural Sciences, and Technology; SSH: Social Sciences and Humanities.

	LS	MINT	SSH	Total
**Reviewers**	**15,006 (100%)**	**15,441 (100%)**	**8,833 (100%)**	**39,280 (100%)**
**Gender of Reviewer**				
Male	11,525 (76.8%)	13,555 (87.8%)	5,433 (61.5%)	30,513 (77.7%)
Female	3,481 (23.2%)	1,886 (12.2%)	3,400 (38.5%)	8,767 (22.3%)
**Country of Affiliation**				
United States of America	3,783 (25.2%)	3,371 (21.8%)	2,051 (23.2%)	9,205 (23.4%)
Germany	1,187 (7.9%)	1,443 (9.3%)	805 (9.1%)	3,435 (8.7%)
Great Britain and N. Ireland	1,072 (7.1%)	1,178 (7.6%)	1,072 (12.1%)	3,322 (8.5%)
France	723 (4.8%)	866 (5.6%)	247 (2.8%)	1,836 (4.7%)
Italy	529 (3.5%)	929 (6.0%)	282 (3.2%)	1,740 (4.4%)
Canada	513 (3.4%)	583 (3.8%)	341 (3.9%)	1,437 (3.7%)
Netherlands	583 (3.9%)	364 (2.4%)	419 (4.7%)	1,366 (3.5%)
Australia	527 (3.5%)	387 (2.5%)	278 (3.1%)	1,192 (3.0%)
Other	2,730 (18.2%)	3,722 (24.1%)	1,344 (15.2%)	7,796 (19.8%)
Unknown	3,359 (22.4%)	2,598 (16.8%)	1,994 (22.6%)	7,951 (20.2%)
**Applicants**	**4,264 (100%)**	**4,077 (100%)**	**3,044 (100%)**	**11,385 (100%)**
**Gender**				
Male	3,074 (72.1%)	3,401 (83.4%)	1,916 (62.9%)	8,391 (73.7%)
Female	1,190 (27.9%)	676 (16.6%)	1,128 (37.1%)	2,994 (26.3%)
**Age**				
Mean (SD)	48.2 (7.5)	46.9 (8.2)	48.2 (7.9)	47.7 (7.9)
**Affiliation**				
Cantonal University	3,383 (79.3%)	1,507 (37.0%)	2,010 (66.0%)	6,900 (60.6%)
ETH Domain	544 (12.8%)	2,150 (52.7%)	183 (6.0%)	2,877 (25.3%)
UAS/UTE	66 (1.5%)	221 (5.4%)	547 (18.0%)	834 (7.3%)
Other	167 (3.9%)	160 (3.9%)	279 (9.2%)	606 (5.3%)
Hospital	86 (2.0%)	2 (0.0%)	3 (0.1%)	91 (0.8%)
Unknown	18 (0.4%)	37 (0.9%)	22 (0.7%)	77 (0.7%)
**Academic Rank**				
Full Professor	1,098 (25.8%)	1,173 (28.8%)	1,254 (41.2%)	3,525 (31.0%)
Associate Professor	858 (20.1%)	534 (13.1%)	468 (15.4%)	1,860 (16.3%)
Assistant Professor	416 (9.8%)	446 (10.9%)	312 (10.2%)	1,174 (10.3%)
UAS/UTE Professor	27 (0.6%)	84 (2.1%)	328 (10.8%)	439 (3.9%)
Senior Postdoc/Group Leader	1,550 (36.4%)	1,540 (37.8%)	599 (19.7%)	3,689 (32.4%)
Other Professorship	290 (6.8%)	274 (6.7%)	76 (2.5%)	640 (5.6%)
Unknown	25 (0.6%)	26 (0.6%)	7 (0.2%)	58 (0.5%)

About a quarter of these reviews were submitted by scholars based in the United States of America (23.4%), followed by Germany (8.7%), Great Britain and Northern Ireland (8.5%), and France (4.7%). In line with SNSF policy, few reviews were submitted by experts from a Swiss institution. For 7,951 (20.2%) reviews, information on the reviewer’s country was not available. There were similar gender differences for applicants. Few women applied for funding in MINT (16.6% female applicants), while LS and SSH had more female applicants (27.9% and 37.1% female, respectively). The mean age of applicants was 47.7 years, and most (60.6%) worked at a Swiss cantonal university, followed by applicants based in the ETH domain (25.3%), i.e., at one of the two Federal Institutes of Technology (ETH Zurich or EPFL Lausanne) or at one of the four ETH research institutions. In MINT, but not in LS or SSH, applications from ETH dominated (Table 1). Almost one in three applicants was a Full Professor (31.0%), followed by Associate Professors (16.3%) and Assistant Professors (10.3%). About a third of applicants were senior postdocs or group leaders (32.4%).

Although the dataset was drawn exclusively from the SNSF, it spanned a wide range of scientific disciplines, included reviewers from many countries, and showed broadly proportional gender distributions among both reviewers and applicants. Together, these features support the relevance of our findings beyond a single disciplinary or national setting. In addition, the SNSF’s evaluation criteria align with widely used international research assessment practices, including commitments associated with the San Francisco Declaration on Research Assessment (DORA) [30] and the adoption of narrative CV formats [31].

### Human annotation of content

We combined human annotations of randomly sampled review sentences with fine-tuned transformer-based machine learning models. After preliminary annotation exercises and various inter-coder reliability tests, we finalized an annotation codebook [32]. Sentences could be assigned to zero, one, or multiple content categories.

Four annotators participated in the study. All had academic backgrounds and experience with peer review, and the team included both native and non-native English speakers, reflecting the international pool of reviewers who submit reports to the SNSF. Each annotator signed a contract prior to the annotation task, which constitutes written informed consent. The contract set out the study’s objectives, the annotators’ roles, and included a confidentiality agreement.

Annotators were trained to recognize that evaluation criteria may be expressed differently across research domains; for example, methodological discussions can differ substantially between the humanities and the natural sciences. They were instructed to assign labels based on conceptual meaning rather than specific terminology, thereby accounting for disciplinary variation in evaluative language.

We implemented several measures to ensure high-quality annotations. The annotation codebook was developed through multiple rounds of piloting and refinement. To reduce individual bias, each sentence was independently labeled by three annotators, and final classifications were determined by majority vote. After each annotation round, we systematically analyzed patterns of agreement and disagreement and used these insights to iteratively improve the guidelines. We also calculated full agreement rates (i.e., cases where all three annotators assigned identical labels) for each category. Across the six categories used in this paper, full agreement ranged from 74% to 90% (mean: 83%).

The annotated dataset of 3,000 sentences constituted a representative sample from a corpus of over 1.3 million sentences, while providing a sufficient amount of training data for fine-tuning the transformer models. An ablation study showed that additional training data beyond 2,000 sentences yielded only marginal improvements in classification accuracy [32]. For most categories, particularly those with balanced class distributions, high classification accuracy was achieved even with smaller samples. This is typical for fine-tuning transformer models, which can achieve strong performance with relatively modest amounts of task-specific training data [33], as also documented in related empirical studies [17,34,35].

To investigate content, we focused on the four SNSF evaluation criteria – (i) Track Record; (ii) Relevance, Originality, Topicality; (iii) Suitability of Methods; and (iv) Feasibility of Project – as well as the sentiment of peer review reports (Positive and Negative).

### Classification using transformer models

We fine-tuned six separate transformer machine learning models for a binary text classification task, one for each content category. We evaluated classification accuracy by splitting the 3,000 annotated sentences into training (2,000), validation (500), and test (500) sets, repeating the train-validation split five times within a cross-validation schema to obtain a more robust estimate of validation error that is less sensitive to any single split [36]. The final models were then fine-tuned using the full set of 2,500 sentences and evaluated on the untouched test set of 500 sentences. Among multiple tested pre-trained transformer models, fine-tuning the SPECTER2 model [37,38] for each category yielded the best performance as measured by the macro-averaged F1 score, both regarding cross-validation as well as test error.

We opted for fine-tuning a SPECTER2 transformer model for several reasons. First, it was pre-trained on scientific texts, making it well-suited to peer review reports [35,39–41]. Second, it supports full transparency and reproducibility: we release our fine-tuned models on Hugging Face (https://huggingface.co/snsf-data), enabling other funding agencies to apply them to their own data. Third, in our comparisons SPECTER2 outperformed a few-shot approach using a large language model (Llama 3 8B [42]), especially for imbalanced categories [32], consistent with evidence that fine-tuned smaller models could match or exceed zero-shot LLM approaches on targeted classification tasks [43]. Finally, SPECTER2 is computationally efficient and can be run locally, avoiding reliance on proprietary services and reducing privacy risks when analyzing confidential review texts.

Overall, our approach prioritizes performance and practical utility. BERT-based models are well established in peer review text analysis [21,44], and our broader model comparison shows that fine-tuned transformers offer a strong balance of accuracy, reproducibility, and computational efficiency [32].

Fine-tuning was conducted locally, without internet access, to eliminate any risk of data leakage or network interference. The macro F1 scores, which account for class imbalances and are frequently used to evaluate out-of-sample performance [45], ranged from 0.79 (Suitability of Methods) to 0.91 (Track Record). These scores were in line with, or higher than, those reported in previous attempts to identify content in peer reviews [17]. S1 Table in S1 File reports the performance metrics for the six machine learning models. Okasa et al. [32] provide further details.

### Predictive terms of content categories

To identify the most characteristic terms for each content category, we conducted keyness analyses using $\chi^{2}$ tests, comparing word frequencies in sentences where a given characteristic was present (target group) with those where it was absent (reference group) [21,46–48]. For each category, we ranked all words by their $\chi^{2}$ values and report the top 25 terms in Table 2. Full details of the approach, including the formula and a worked example, are provided in S1 Text in S1 File.

**Table 2:** Frequent predictive terms for each content category. The results are based on keyness analyses using $\chi^{2}$ tests for each word or multi-word expression, comparing frequencies in sentences where a content characteristic was present (target group) with those where it was absent (reference group). The term “unk” is a placeholder for anonymized names of persons involved in a proposal.

Category	Predictive Terms
***Evaluation Criteria***	
Track Record	unk, expertise, applicant, dr, publications, experience, track_record, field, published, prof, pi, carry, team, journals, papers, professor, university, expert, excellent_track_record, phd, research, publication_record, cv, career, excellent
Relevance, Originality, Topicality	originality, original, novel, timely, topical, topicality, scientific_relevance, topic, relevant, relevance, highly_relevant, innovative, understanding, interesting, novelty, potential, discipline, important, field, scientifically_relevant, exciting, project, interest, proposed_project, broader_impact
Suitability of Methods	methods, suitable, appropriate, suitability, proposed_methods, methodology, methods_proposed, well_suited, method, suited, chosen_methods, answering, adequate, questions_set, methods_chosen, feasible, choice, approach, proposed_methodology, chosen, sample_size, answer, methods_described, use, used
Feasibility of Project	feasible, feasibility, milestones, timeline, project, workload, budget, schedule, personnel, planned_duration, proportionate, reasonable, given_time, achievable, time_frame, time, realistic, timeframe, planned, available_resources, highly_feasible, ambitious, milestones_set, reached, timing
***Sentiment***	
Positive	field, project, excellent, unk, expertise, original, applicant, good, overall, proposed_project, relevant, timely, carry, outstanding, highly_relevant, feasible, team, research, strong, topical, interesting, track_record, proposed_research, innovative, originality
Negative	lack, however, unclear, clear, difficult, little, missing, weakness, limited, concern, bit, seems, unfortunately, seem, somewhat, vague, lacks, weaknesses, lacking, details, proposal, questionable, concerns, description, although

Sentences labeled as *Track Record* often contained words such as “unk” (a placeholder for anonymized “unknown” names of persons), “expertise,” “applicant,” “publications,” “professor,” and “cv,” reflecting references to professional background and productivity. *Relevance, Originality, Topicality* included terms like “original,” “innovative,” “highly relevant,” “scientific relevance,” and “broader impact,” indicating novelty and contribution. *Suitability of Methods* was characterized by words such as “proposed methods,” “methodology,” and “sample size,” emphasizing methodological appropriateness. *Feasibility* included “feasibility,” “timeline,” “planned duration,” and “available resources.” *Positive* sentiment featured terms like “excellent,” “original,” and “innovative,” while *Negative* sentiment included “lack,” “unclear,” and “weakness,” reflecting approval or critique.

### Statistical analysis

After applying the six classifiers to each sentence to detect the presence or absence of the four evaluation criteria as well as positive and negative sentiment, we aggregated the data to the review level to calculate the prevalence of each content category. Because the categories were not mutually exclusive, a single sentence may be assigned to multiple categories, which could result in combined prevalences exceeding 100%. This approach, consistent with prior work [21], enabled comparisons of the relative emphasis placed on different aspects within peer review reports.

Given the clustered nature of the data, where each proposal received multiple reviews, we employed a mixed-effects regression model to examine the relationship between gender, discipline, and review content [49]. In this model, the effects of reviewer gender, applicant gender, discipline, and control variables were treated as fixed effects, while random intercepts were introduced at the proposal level to account for within-proposal correlation. Formally, we specified the following linear model:


$$\begin{array}{cc} Y_{ij}=\; & \alpha +\gamma G_{ij}^{R}+\delta G_{j}^{A}+\theta D_{j}+\beta X_{ij}+U_{j}+\varepsilon_{ij}\\ & U_{j}\sim 𝒩(0,\sigma_{u}^{2})\\ & \varepsilon_{ij}\sim 𝒩(0,\sigma^{2}) \end{array}$$

where $Y_{ij}$ represents the text-derived outcome for review *i* of proposal *j*. α is the overall intercept, γ is the coefficient for reviewer *i*’s gender $G_{ij}^{R}$ evaluating proposal *j*, δ is the coefficient for applicant *j*’s gender $G_{j}^{A}$, θ is the coefficient for proposal *j*’s discipline $D_{j}$, and β is a vector of fixed coefficients related to the vector of control variables $X_{ij}$, while $U_{j}$ is the random intercept for proposal *j* with variance $\sigma_{u}^{2}$. The unobserved error term is denoted by $\varepsilon_{ij}$ with variance $\sigma^{2}$. Our primary interest lies in the estimation of the coefficients γ, δ, and θ, which measure the gender and disciplinary differences.

We used one-hot encoding for the research domain variable $D_{j}$, leaving out the LS research domain as the reference category. The models were adjusted for potential confounding variables. These included funding call-specific factors (e.g., call deadline, with two calls per calendar year) and applicant-level characteristics, such as career stage, prior experience with SNSF (whether the applicant had previously received or submitted grants), requested project duration, research institution type, and type of professorship. We also accounted for proposal-level attributes, including the presence of multiple applicants, resubmission status, collaboration under a lead agency agreement, and classification as use-inspired research. At the review level, the models controlled for whether the reviewer reported the proposal to be within their area of expertise, the length of the review, the number of reminders sent, and the reviewer’s geographic region. Finally, demographic variables, including applicants’ age, were included.

All variables used in the regression analysis are detailed in S2 Table in S1 File. We conducted multiple robustness tests on control variables, model specifications, model assumptions, and also incorporated the overall grade the reviewers assigned to proposals in our regression models (S3 Text, S2 Fig, S4–S6 Tables in S1 File). Until 2021, reviewers graded the proposal on a six-point scale, and a nine-point scale thereafter. We therefore rescaled the reviews from a nine-point range to a 1–6 range and used this standardized measure.

## Results

We present the results in several steps, starting with descriptive statistics on the differences in the length of reviews across disciplines, the gender of reviewers, and the gender of the applicants. Then, using descriptive and mixed-effects regression analyses, we assess how content related to the SNSF evaluation criteria and how the sentiment of peer review reports differ across disciplines and gender. Moreover, we report on the role of the review score and on other robustness analyses.

### Review length

**Fig 1:**
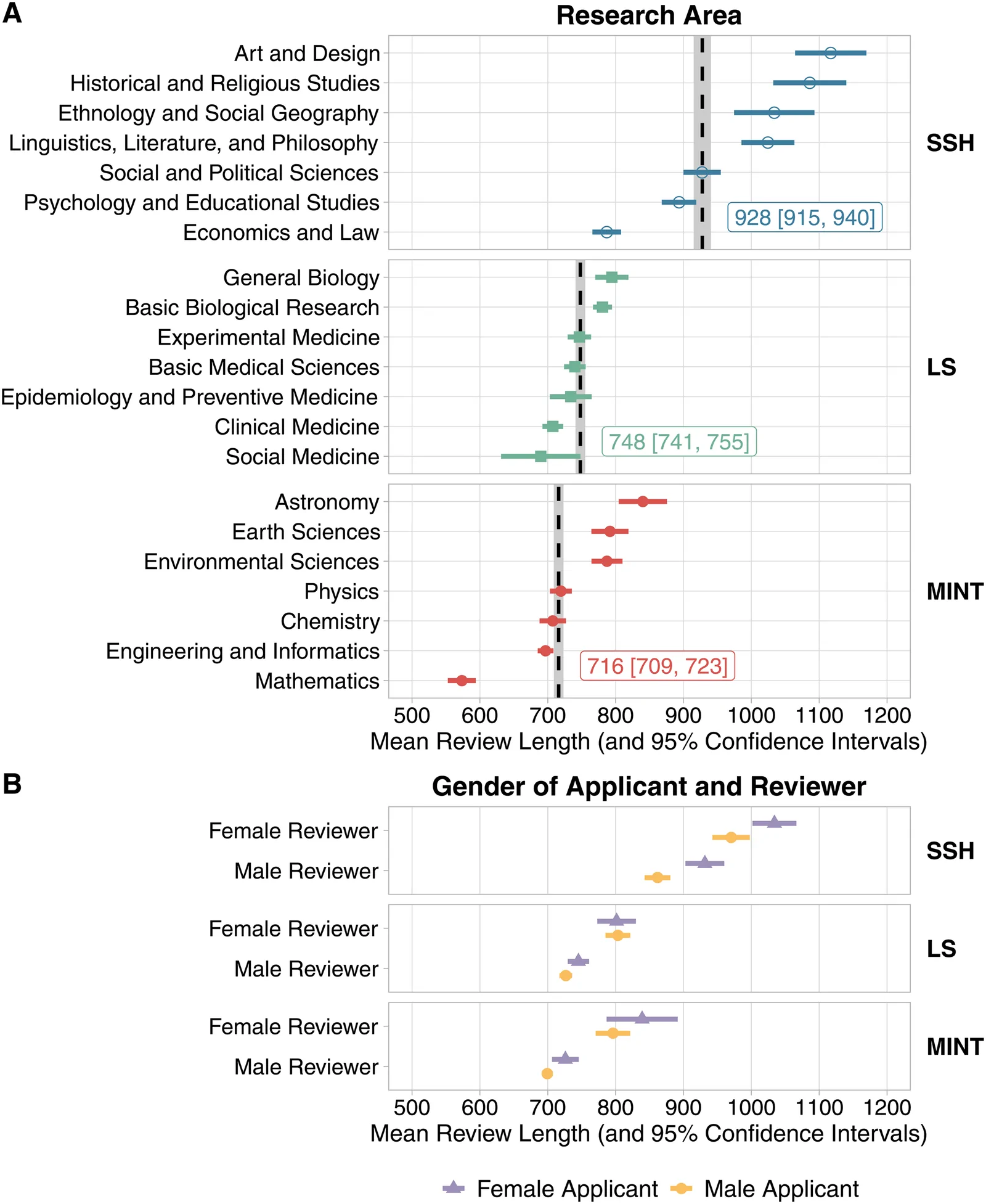
Average review length conditionally on the research domain (panel A) and gender of applicants and reviewers (panel B). Dots show the mean length (in words), horizontal bars the 95% confidence intervals. SSH: Social Sciences and Humanities; LS: Life Sciences; MINT: Mathematics, Informatics, Natural Sciences, and Technology.

The word count of reviews differed across the 21 SNSF disciplines (Panel A of Fig 1, see S7 Table in S1 File for further details on disciplinary groups). With a mean of 928 words, reviews in the SSH were considerably longer than reviews in the LS and MINT. We observed further differences within each research domain. With 1,117 words, reviews in the discipline Art and Design received the longest, closely followed by Historical and Religious Studies (1,086 words). In contrast, the reviews submitted in Economics and Law were shorter than 800 words. The mean length in LS varied from 690 words in Social Medicine to 794 words in General Biology. By far the shortest reviews were submitted for proposals in Mathematics, with a mean review length of 573 words.

Female reviewers submitted longer reviews than male reviewers in all three research domains (Panel B of Fig 1). The difference was largest in the SSH. In SSH and MINT, but not in the LS, female applicants received, on average, longer reviews from both female and male reviewers. This difference was again most pronounced for the SSH. The gender of reviewers was more strongly associated with review length than the gender of applicants (S3 Fig in S1 File).

### Review content

**Fig 2:**
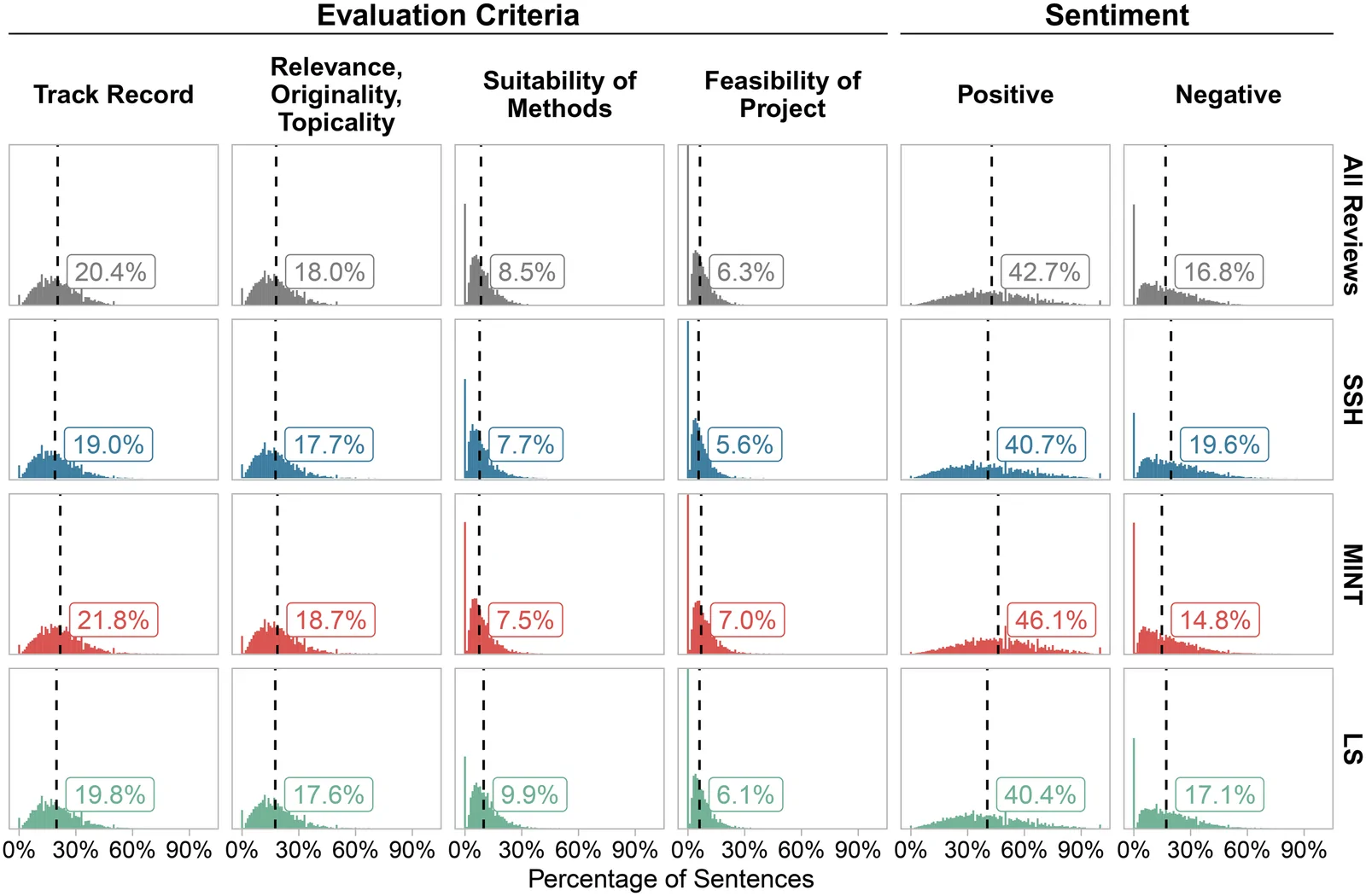
Distribution of sentences in peer review reports allocated to four evaluation criteria and two sentiment categories for the full sample and by research domain. A sentence could be allocated to no, one, or several categories. Vertical dashed lines and numbers in graphs depict the mean percentage of sentences after aggregating them to the review level. SSH: Social Sciences and Humanities; MINT: Mathematics, Informatics, Natural Sciences, and Technology; LS: Life Sciences.

The applicant’s *Track Record* and the *Relevance, Originality, and Topicality* of the proposal were the most frequently addressed criteria. Across all reviews, the track record accounted for an average of 20.4% of sentences, while 18.0% discussed the relevance, originality, and topicality of the proposed research (Fig 2). The comparison across research fields showed that reviews in MINT place greater emphasis on both the applicant’s track record and the scientific relevance of the proposal. *Suitability of Methods* was discussed in 8.5% of all sentences, with LS placing comparatively more emphasis on this criterion (9.9% of sentences). *Feasibility* was addressed in 6.3% of sentences overall, with the SSH allocating slightly fewer sentences (5.6%) to this criterion than LS (6.1%) and MINT (7.0%). Fig 2 also showed that *Positive Sentiment* was more prevalent than *Negative Sentiment* in peer review reports. On average, 42.7% of review sentences expressed positive sentiment, while only 16.8% were negative. Notably, reviews in the MINT fields were more positive on average than those in the other two domains.

Further stratifying the analysis by 21 academic disciplines showed that Mathematics (25.6%), Physics (23.5%), Astronomy (22.5%), and Chemistry (22.4%) placed the most emphasis on the applicant’s track record (S4 Fig in S1 File). In contrast, disciplines within the social and health sciences, such as Social and Political Sciences, had the lowest prevalence (17.6%). For relevance, originality, and topicality, the differences across disciplines were smaller, suggesting a relatively consistent application of this criterion across disciplines (S5 Fig in S1 File). In contrast, the suitability of the methods showed larger differences (S6 Fig in S1 File), with the LS, particularly Epidemiology and Preventive Medicine and Social Medicine, emphasizing this criterion (11.8% and 11.4% of review sentences, respectively), compared to under 5.5% in humanities-oriented SSH disciplines, such as Historical and Religious Studies. Finally, the feasibility of the proposed research (S7 Fig in S1 File) was discussed slightly more in MINT and LS disciplines than in SSH.

**Fig 3:**
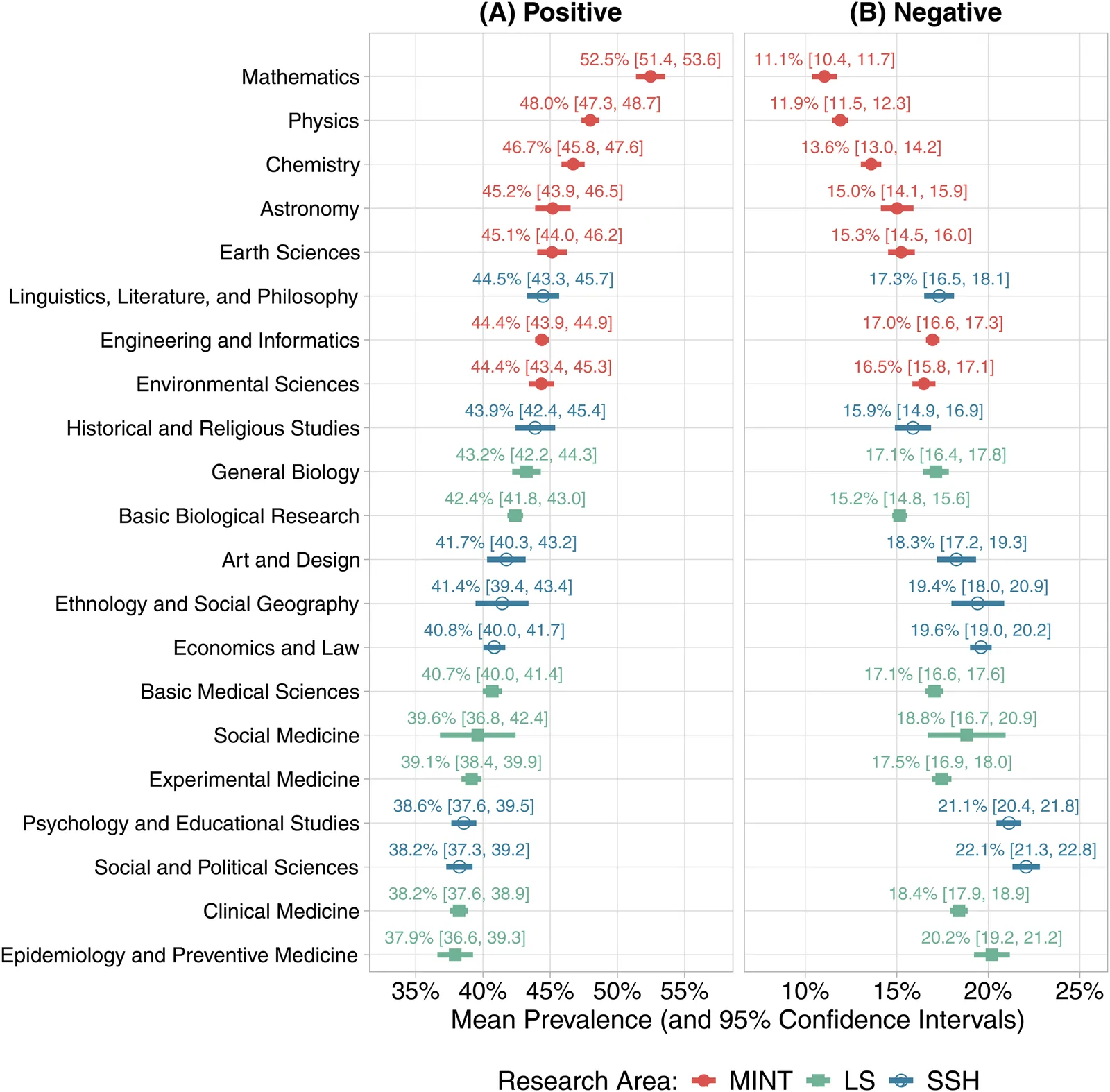
Average prevalence of positive (A) and negative (B) statements (% of review sentences) by discipline. Horizontal bars show 95% confidence intervals. Separate horizontal axis limits are used to allow for comparisons within each content category. Corresponding figures showing the distribution of each category across all disciplines are provided in S4–S7 Figs in S1 File.

There were substantial differences in the tone of peer review reports across the 21 disciplines. Reviews in MINT disciplines were more positive on average than those in SSH and LS (Fig 3). For example, in Mathematics, more than half of the sentences in an average review (52.5%) were positive, compared to only 37.9% in Epidemiology and Preventive Medicine. We observed similar patterns for the prevalence of negative sentences. Reviews in SSH disciplines, in particular Social and Political Sciences as well as Psychology and Educational Studies, were more negative (22.1% and 21.1%, respectively) than in the MINT disciplines. For example, only 11.1% of sentences in reviews of Mathematics and 11.9% of Physics proposals were negative.

### Regression models

The results from mixed-effects regression models, adjusted for length of review and potential confounders, are shown in Table 3, with coefficients indicating percentage point differences relative to the reference category. Overall, the evaluation criteria were covered more comprehensively by female reviewers than by male reviewers. Further, female reviewers made fewer negative comments than male reviewers (difference: –0.98). Focusing on the gender of the applicants, female applicants received slightly more methodological comments than male applicants (difference: 0.43), with little evidence of differences for the other evaluation criteria. Further, female applicants received both slightly more positive and fewer negative remarks (difference: 0.50 and –0.46 percentage points, respectively).

**Table 3:** Predicting peer review content. Coefficients from mixed-effects models show percentage point differences in prevalence relative to reference category. Models include control variables listed in S2 Table in S1 File and random intercepts for proposal IDs. 95% confidence intervals in square brackets. Analysis based on 39,280 peer review reports on 11,385 proposals. MINT: Mathematics, Informatics, Natural Sciences, and Technology; SSH: Social Sciences and Humanities; LS: Life Sciences.

	Evaluation Criteria				Sentiment	
	Track Record	Rel., Orig., Topic.	Methods	Feasibility	Positive	Negative
Reviewer: Female (ref.: Male)	1.00	0.76	0.48	0.60	0.59	−0.98
	[0.74, 1.26]	[0.52, 1.01]	[0.31, 0.64]	[0.46, 0.74]	[0.15, 1.04]	[–1.31, –0.65]
Applicant: Female (ref.: Male)	0.22	−0.05	0.43	0.03	0.50	−0.46
	[–0.05, 0.48]	[–0.31, 0.20]	[0.25, 0.61]	[–0.12, 0.17]	[0.02, 0.97]	[–0.82, –0.10]
Domain: MINT (ref.: LS)	1.82	0.61	−2.19	0.74	4.65	−1.72
	[1.52, 2.11]	[0.33, 0.88]	[–2.39, –1.99]	[0.58, 0.90]	[4.13, 5.17]	[–2.12, –1.33]
Domain: SSH (ref.: LS)	−0.77	0.81	−2.09	−0.44	2.71	1.59
	[–1.09, –0.45]	[0.50, 1.11]	[–2.31, –1.87]	[–0.61, –0.26]	[2.14, 3.28]	[1.16, 2.02]

There were notable differences across the three research domains. Compared to LS, proposals in the MINT and SSH disciplines placed less emphasis on the suitability of methods (difference around –2 percentage points), and more emphasis on the relevance and originality of the research (Table 3). Reviews of MINT projects emphasized the track record of applicants and the feasibility of the research more than LS reviewers (differences: 1.82 and 0.74, respectively), whereas the opposite was the case for SSH (differences: –0.77 and –0.44, respectively). MINT proposals received substantially more positive comments than LS projects (difference 4.65 percentage points) and fewer negative comments (−1.72), whereas SSH proposals received both more positive and more negative comments (Table 3).

When including the overall grades into the model (S5 Table in S1 File), higher grades were associated with an increase in the prevalence of text on all evaluation criteria except for the methods, which received less attention (difference per grade point increase: –0.45). As could be expected, positive comments were strongly associated with higher grades (9.60 percentage points per grade point increase), whereas reviews with higher grades contained fewer negative comments (–8.00). While grades predicted some evaluation criteria and sentiment, the observed differences across research areas and the gender of reviewers and applicants remained.

## Discussion

This analysis of nearly 40,000 peer review reports shows systematic variation in how evaluations are written across disciplines and genders. Compared to LS reviews, SSH reviews were longer and more critical, with less emphasis on track record, while MINT reviews were shorter, more positive, and focused more on track record. Female reviewers wrote longer and more positive reviews that more closely aligned with the evaluation criteria. Further, female applicants received reviews with slightly more methodological content and a more positive tone. Our findings suggest that gender and disciplinary conventions shape the content of grant peer review reports and demonstrate that review texts contain information not captured by the overall scores assigned by reviewers.

Few previous studies have examined peer review texts from funding agencies, focusing instead on numerical scores. A 1997 Swedish study found that female applicants for postdoctoral fellowships had to demonstrate greater productivity than males to receive equivalent scores [50]. More recent SNSF analyses revealed disciplinary variation in scores, with some fields (e.g., mathematics, physics, history) scoring higher than medicine or psychology [11]. Male reviewers gave higher scores to male applicants, whereas no similar bias was observed among female reviewers [11]. In line with our results, Luo et al. [19] found that sentiments correlate with review scores and the ranking of proposals, particularly in low-success-rate programs where negative sentiment strongly predicted rejection.

Journals have moved toward more open processes, with peer reviews often published and reviewer activity recognized as contributions to science, thus facilitating research on journal peer review [51,52]. Studies of journal peer review indicate both parallels and contrasts with the present work. Analyses of nearly 500,000 journal reviews [53] found that reviewers in the social sciences and economics emphasized methodological rigor more than those in other fields [54]. In contrast, our study suggests that reviewers for SSH and MINT proposals focused less on the suitability of methods than reviewers in the LS. A study of peer review in neuroscience journals found female first authors received less polite feedback, whereas female last authors received more favorable reviews [18]. The latter aligns with our findings, as last authors would more likely be senior investigators in LS and thus more likely eligible to apply for SNSF Project Funding, and female applicants received more positive reviews in our study.

Strengths of this study include the size and diversity of the dataset, which covers all major disciplines and draws on an international reviewer pool. It is among the first large-scale textual analyses of grant reviews, combining human annotation with transformer-based machine learning models, achieving high classification accuracy [32]. The methodology is transparent, reproducible, and openly shared, avoiding reliance on proprietary systems [55]. Combining computational and regression approaches allowed nuanced insights, and the international reviewer base enhances generalizability to other funding systems. Language detection, fine-tuning, and sentence-level classifications were conducted locally, and all shared data were fully anonymized, with no possibility of reconstructing review texts. All contributors signed data-sharing agreements, and a Data Management Plan ensured compliance.

Our study helps move peer review research beyond describing “deficits” (e.g., low agreement or bias) toward the mechanisms that produce observed patterns. Recent conceptual work argues that peer review is best understood as a set of interrelated practices that generates (i) expert judgments of quality, (ii) allocation decisions under scarcity, and (iii) legitimacy for these decisions within science and to external audiences [56,57]. The disciplinary differences in how criticism and praise are expressed suggest that peer review reflects field-specific evaluative cultures rather than functioning as a uniform measurement instrument: what counts as a “quality signal” may vary across disciplines. Status- and role-based theories further predict that gender can shape competence expectations and standards for evaluating ability [58,59], which may translate into systematic differences in how reviewers describe applicants and frame strengths and weaknesses.

Limitations include the inability to assess how gender and disciplinary differences affected panel discussions or funding outcomes, the restriction to English-language reports, and the observational design, which limits causal inference. External peer review at the SNSF is only one stage in a multi-step evaluation process. Funding decisions are made during panel deliberations, where external reviews are discussed, interpreted, and weighed alongside internal assessments, but these discussions are not available for systematic analysis. We show that review characteristics vary by gender and disciplinary context, yet we cannot assess whether these differences translate into funding disparities. Addressing this question would require research designs that capture panel-level decision-making, which is beyond the scope of this study. In addition, because non-English reports were excluded, our findings apply only to English-language peer review reports. This includes virtually all reports in LS and MINT, but only about two-thirds of reports in SSH (see S3 Table in S1 File). In SSH, the proportion of English-language reports increased from around 55% in 2016 to around 70% or more from 2020 onward, suggesting that any bias introduced by excluding non-English reports was greater in the earlier part of the study period. Nevertheless, the results should be interpreted with caution for SSH, where inclusion of non-English reports might have changed the observed patterns.

The set of potential confounding variables was not exhaustive, which may limit model robustness and explanatory power. For example, we could not directly assess institutional prestige but included applicants’ institution type as a proxy. Because the study is observational, our estimates should be interpreted as conditional associations rather than causal effects. Nevertheless, we adjusted for a range of observable characteristics that could influence both review content and our variables of interest, i.e., reviewer and applicant gender, and proposal discipline. Further, gender was treated as binary and assigned only to the corresponding applicant, potentially oversimplifying the diversity of applicant and team identities. Finally, sentiment analysis may miss subtle or discipline-specific forms of critique or praise [60].

While 2,500 annotated sentences were enough to achieve reliable performance on a 500-sentence test set, the sample remains small, so classifications should be used cautiously. The classifiers should not be used for automated decision-making without human oversight, given the known limitations of large language models, including difficulties in assessing research quality and risks of reproducing biases. Further, our classification models may underestimate rare but meaningful forms of evaluative language specific to particular disciplines. Discipline-specific rhetoric that diverges substantially from the training data may therefore be misclassified. S4 Text in S1 File provides further details on data protection, data sharing, and responsible use.

Our results have practical implications: if disciplines differ in how they express criticism, confidence, or praise, reports may not be directly comparable across fields. This strengthens the case for structured review templates and well-defined criteria that prompt reviewers to address the same dimensions consistently and separate criterion-based judgments from overall sentiment. Mandatory justification fields could further increase transparency and help panels interpret reviews. The evidence on training for grant reviewers remains limited [61]. A randomized trial of automated feedback found little improvement in score agreement [62], indicating that training should focus on how to interpret criteria rather than on scoring patterns. Panel-level calibration may benefit from brief pre-panel discussions of sample applications and benchmarks, supported by written guidance. Finally, large language models could support quality assurance by analyzing review texts over time; for example, monitoring whether some panels provide systematically more negative or less criterion-specific feedback, or whether interventions reduce variation.

## Conclusions

Our study highlights that disciplinary differences and reviewer gender relate to the tone and content of grant peer review reports, with potentially important implications for fairness and consistency in funding decisions. We find that reviewers do address the evaluation criteria, but the focus on these criteria in review reports varies considerably. Similarly, the observation that female reviewers write more positive and detailed evaluations points to gendered communication styles that may affect how funding applications are evaluated. These patterns raise concerns about the comparability of peer reviews across reviewer demographics and disciplines, particularly in multidisciplinary panels. The results underscore the need to account for structural variation in review practices when designing peer review systems, setting scoring guidelines, or training reviewers [63–66]. Interventions such as reviewer calibration, structured review formats, or diversity policies could help reduce unintended disparities and promote more equitable and transparent evaluation processes. Future research should examine how disciplinary and interdisciplinary panels interpret and integrate review reports with differing emphases or sentiment, and how funding decisions are reached.

## Supporting information

S1 File This file contains S1 Text–S4 Text, S1 Table–S7 Table, and S1 Fig–S7 Fig. (PDF)
