# Supplementary material for: Gender and disciplinary differences in grant proposal peer review: Content and sentiment in 39,280 reports
Source: PLoS One. 2026 Sep 15;21(9):e0352900. doi: 10.1371/journal.pone.0352900 (PMC13577508; doi:10.1371/journal.pone.0352900)
Supplement: Supplementary file 1 [file pone.0352900.s001.pdf]

Supporting Information

Gender and disciplinary differences in grant proposal peer review: Content and sentiment in 39,280 reports

Stefan Müller, Gabriel Okasa, Michaela Strinzel,

Anne Jorstad, Katrin Milzow, and Matthias Egger

Corresponding author: Stefan Müller

stefan.mueller@ucd.ie

S1 Table: *Overview of the classification test results.* Table shows Accuracy (Acc.), Balanced Accuracy (Bal. Acc.), the F1 score, Precision (Prec.), Recall (Rec.), and class-specific performance metrics for F1 score, Precision, and Recall for the presence (Lab=1) and absence (Lab=0) of a characteristic. Results are based on six separate fine-tuned transformer models. The metrics can range from 0 to 1 with higher values indicating better model classification. Detailed results and systematic comparisons of various classification approaches are presented in Okasa et al. [1].

Category	Acc.	Bal. Acc.	F1	F1 Lab=1	F1 Lab=0	Prec.	Prec. Lab=1	Prec. Lab=0	Rec.	Rec. Lab=1	Rec. Lab=0
Track Record	0.95	0.92	0.91	0.86	0.97	0.90	0.82	0.98	0.92	0.89	0.96
Rel., Orig., Topic.	0.92	0.86	0.86	0.76	0.95	0.85	0.75	0.95	0.86	0.78	0.95
Suitability of Methods	0.94	0.77	0.79	0.62	0.97	0.82	0.69	0.96	0.77	0.56	0.98
Feasibility of Project	0.97	0.85	0.87	0.76	0.99	0.90	0.81	0.98	0.85	0.71	0.99
Positive	0.90	0.90	0.89	0.87	0.92	0.89	0.84	0.94	0.90	0.90	0.90
Negative	0.91	0.84	0.83	0.71	0.94	0.81	0.67	0.95	0.84	0.75	0.93

Variable name	Description
gender_reviewer	gender of reviewer (male/female)
gender_applicant	gender of (corresponding) applicant. Note: gender (male/female) missing for three applications
ResearchArea	research domain (LS/MINT/SSH)
CallEndDate	submission deadline of call
DurationRequestedMonth	requested duration of the project as submitted by applicant (in months)
IsHasPreviousProjectApproved	indicates if the applicant has previously received a grant from the SNSF
IsHasProjectPartners	indicates if the proposal has been submitted with project partners
IsHasPreviousProjectRequested	indicates if the applicant has previously applied for a grant from the SNSF
IsMultiApplicant	indicates whether application has several applicants. Note: variables on the applicant always rely on characteristics of the corresponding applicant
IsLeadAgencySNF	indicates if the submission is a collaboration with a foreign funder, handled by the SNSF
IsResubmission	indicates if proposal is a resubmission of a previously rejected grant proposal
IsUseInspired	indicates if the project is use-inspired basic research
MatchToApplicationTopic	question whether application topic is within the reviewer's self-reported area of specialization or within reviewer's wider discipline
ntoken_review	length of review reports (in words)
ReminderCount	number of reminders sent to reviewer
ResearchInstitutionAtProjectStartDateType	research institution type of the applicant
ResponsibleApplicantAgeAtSubmission	age of applicant at time of submission (invalid ages for 59 applications due to data entry errors by the applicants; removed from sample before analysis)
ResponsibleApplicantProfessorshipType	professorship type of the applicant
ReviewerCountry	country of reviewers' institution
ReviewerDegree	highest achieved academic degree of the reviewer
reviewer_region	region of reviewer (recoded from ReviewerCountry)

S2 Table: *Overview of variables used in analysis*

S1 Text Keyness analysis methodology

We conducted so-called keyness analyses using chi-squared (χ^2) tests to identify the most characteristic terms for each content category. We performed separate analyses for each of the six content categories (*Track Record*; *Relevance, Originality, Topicality*; *Suitability of Methods*; *Feasibility of Project*; *Positive*; *Negative*). For each category, we split the corpus into sentences where the characteristic was present (target group) and sentences where it was absent (reference group). Since sentences could be assigned to multiple categories, the same sentence might appear in the target group for one category and the reference group for another.

Within each analysis, we calculated the chi-squared statistic for every word and previously identified multi-word expression, comparing its frequency in the target group with its frequency in the reference group. The chi-squared statistic measures how much observed word frequencies deviate from expected frequencies under independence. The formula is:

$$\chi^2 = \frac{N \times (ad - bc)^2}{(a+b)(c+d)(a+c)(b+d)}$$

where a is the frequency of the word in the target group, b is its frequency in the reference group, c is the frequency of all other words in the target group, d is the frequency of all other words in the reference group, and N is the total word frequency across both groups. Higher χ^2 values indicate stronger association between a word and the target category. For each category, we ranked all words by their χ^2 values.

To illustrate the approach, we provide a simple example. Consider the word 'methodology' when comparing sentences about the *Suitability of Methods* (n = 10,000 words, including 'methodology' 50 times) with other sentences (n = 90,000 words, including 'methodology' 30 times). The chi-squared statistic is:

$$\chi^2 = \frac{100{,}000 \times (50 \times 89{,}970 - 30 \times 9{,}950)^2}{(80)(99{,}920)(10{,}000)(90{,}000)} = 245.20$$

Keyness values were calculated using the `textstat_keyness()` function from the `quanteda.textstats` package (version 0.97) in R (version 4.4.2), with `measure = "chi2"`. This implementation does not apply

Yates's continuity correction, which is standard practice in corpus linguistics for large samples [2].

S2 Text Difference in review length across languages and prevalence of English reviews

This section examines two aspects of language use in grant peer reviews. First, we compared review length across the four languages in the corpus. Second, we reported the prevalence of English-language reviews across reviewer subgroups (gender, country of affiliation, and submission year) both for the full sample and separately by research area. Because the automated classifiers for sentiment and content are limited to English text, the prevalence figures establish the share of reviews that feed into the downstream analyses and allow us to assess whether non-English reviewing was concentrated among particular reviewer groups.

The sample for this analysis was created as follows: Starting from 44,026 reviews, we applied four sequential filters to arrive at the analytic sample. First, we removed 203 reviews identified as written in multiple languages, as switching between languages within a review would confound both the prevalence calculation and the length comparison.[1] Second, we dropped three reviews for which language detection returned missing values, caused by empty or very short text fields. Third, we excluded three reviews linked to applicants with implausible ages (below 18 or above 100 years at the time of submission). Fourth, we removed eleven reviews where the responsible applicant's gender is recorded as unknown. These four steps reduce the sample by 220 reviews, yielding a final analytic sample of 43,806 reviews.

S1 Fig shows that review length varies considerably across the four languages in the corpus, though only English reviews feed into the classifiers. At an average of 776 tokens, English reviews were shorter than reviews written in other languages. German and Italian reviews were around 23% and 24% longer, respectively, averaging 958 and 964 tokens. French reviews were the longest at 1,072 tokens, roughly 38% above the English average. As the numbers beside the bars indicate, 90.1% of reviews (39,469 of 43,806) were written in English.[2] The finding that English reviews are shorter on average than those written in other languages aligns with research showing that translating English text into German or French increases the word count [3].

[1] Reviews flagged as multi-language were identified at the sentence level.

[2] The regression models in the main paper were estimated on a slightly smaller sample of 39,280, as we restricted the regression analyses to reviews with complete covariate data to ensure consistency across all analyses. The 189 excluded

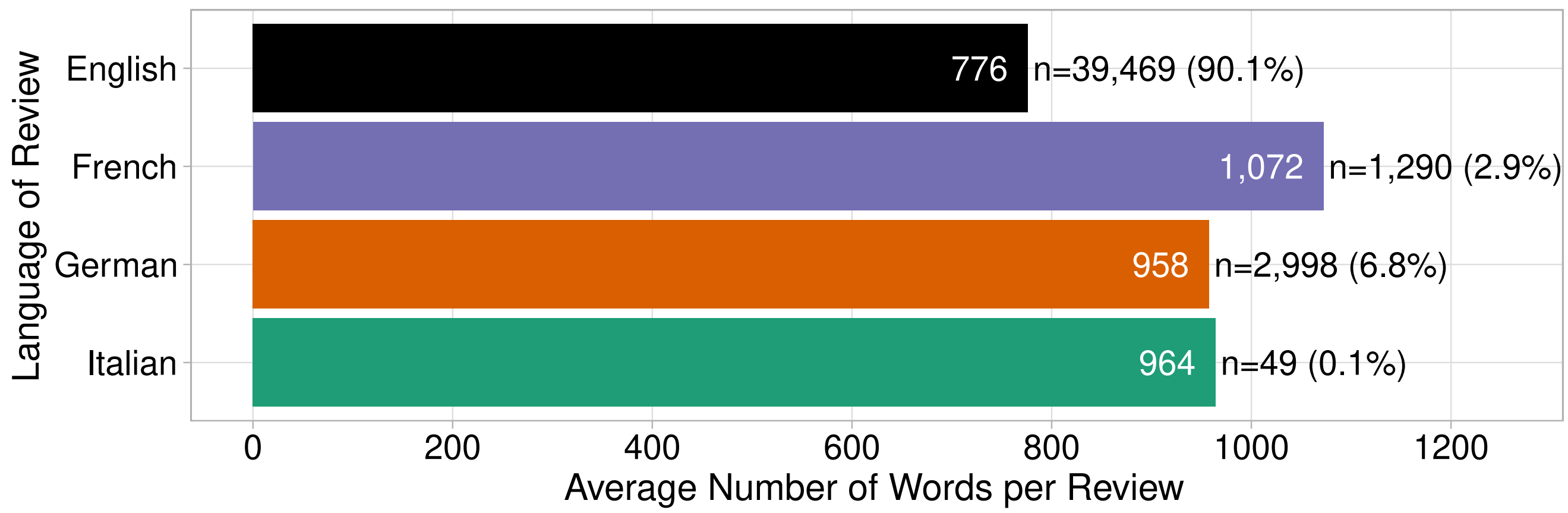

S1 Fig: *Comparison of average review length across languages.* The difference in the number of English reviews in this analysis and the sample used for the empirical analysis stems from missing covariates for some of the reviews used in the regression analysis.

S3 Table reports the prevalence of English-language reviews broken down by reviewer gender, country of affiliation, and submission year, separately for each of the three research areas and for the full sample. English-language reviews dominated overall. In Life Sciences and MINT, almost all reviews were in English whereas in SSH two thirds of reports were in English. English-language use also varied by reviewer characteristics. It was lower among female reviewers overall because more female reviewers were in SSH. In LS and MINT, over 99% of all reviews by female reviewers were submitted in English. English reviews were also lower for SSH reviewers from Germany and France than for reviewers from English-speaking countries. Over time, the proportion of English-language reviews increased to over 90%, mainly because English use rose in SSH.

S3 Text Further analyses

We assessed the robustness of our results along three dimensions: *model specification*, *control variables* and *model assumptions*. First, to check the sensitivity of our results to model specification, we additionally test for an interaction between reviewer and applicant gender and estimate the following model:

$$Y_{ij} = \alpha + \gamma G^R_{ij} + \delta G^A_j + \zeta(G^R_{ij} \times G^A_j) + \theta D_j + \beta X_{ij} + U_j + \varepsilon_{ij}$$

where the coefficient ζ measures the gender interaction effect. When including an interaction term in

reviews are present in the prevalence figures but lack some of the control variables required for the regression.

S3 Table: *The prevalence of English-language reviews by reviewer characteristics.* Each cell shows the count of all reviews (English and other languages combined) and the percentage of English-language reviews within that category.

	LS	MINT	SSH	Total
**Reviews**	**15,162 (99.6%)**	**15,680 (98.9%)**	**12,964 (68.4%)**	**43,806 (90.1%)**
**Gender of Reviewer**				
Female	3,507 (99.7%)	1,915 (99.2%)	5,164 (66.1%)	10,586 (83.2%)
Male	11,655 (99.5%)	13,765 (98.8%)	7,800 (69.9%)	33,220 (92.3%)
**Country of Affiliation**				
United States of America	3,813 (100.0%)	3,391 (99.9%)	2,083 (99.3%)	9,287 (99.8%)
Germany	1,220 (98.1%)	1,520 (95.4%)	2,310 (35.2%)	5,050 (68.5%)
Great Britain and N. Ireland	1,078 (100.0%)	1,183 (100.0%)	1,084 (99.4%)	3,345 (99.8%)
France	732 (99.0%)	908 (95.7%)	857 (28.8%)	2,497 (73.7%)
Italy	535 (100.0%)	939 (100.0%)	340 (83.2%)	1,814 (96.9%)
Canada	517 (100.0%)	586 (99.5%)	487 (70.4%)	1,590 (90.8%)
Netherlands	585 (100.0%)	368 (99.7%)	428 (98.1%)	1,381 (99.3%)
Australia	527 (100.0%)	388 (100.0%)	278 (100.0%)	1,193 (100.0%)
Other	2,759 (99.9%)	3,749 (99.9%)	1,670 (81.3%)	8,178 (96.1%)
Unknown	3,396 (99.1%)	2,648 (97.8%)	3,427 (57.8%)	9,471 (83.8%)
**Submission Year**				
2016	881 (99.7%)	917 (98.5%)	933 (56.9%)	2,731 (84.7%)
2017	1,808 (99.5%)	1,897 (98.2%)	1,559 (62.0%)	5,264 (87.9%)
2018	1,982 (99.4%)	2,052 (98.5%)	1,664 (65.3%)	5,698 (89.1%)
2019	1,948 (99.3%)	2,020 (98.8%)	1,815 (68.8%)	5,783 (89.5%)
2020	2,264 (99.5%)	2,510 (99.0%)	2,129 (71.3%)	6,903 (90.7%)
2021	2,444 (99.6%)	2,474 (99.2%)	2,072 (73.5%)	6,990 (91.7%)
2022	2,520 (99.8%)	2,543 (99.2%)	1,801 (72.6%)	6,864 (92.4%)
2023	1,315 (99.8%)	1,267 (99.2%)	991 (69.4%)	3,573 (91.2%)

the model (female reviewer × female applicant), we find that when both the reviewer and applicant are female, reviews include more positive and fewer negative comments. In contrast, there was little evidence for interactions between the genders of reviewers and applicants for the evaluation criteria (S4 Table). Stratifying this analysis by research area, it becomes clear that these results are driven by the LS (S2 Fig).

Second, we check the robustness by enlarging the set of control variables by adjusting for the overall grade assigned by the reviewer to a proposal. We rescale grades as described in the Methods section of the main paper. We estimate the following equation:

$$Y_{ij} = \alpha + \gamma G_{ij}^{R} + \delta G_{j}^{A} + \theta D_j + \vartheta Z_{ij} + \beta X_{ij} + U_j + \varepsilon_{ij}$$

where ϑ is the coefficient for the grade Z_{ij} given by reviewer i to proposal j. The results of the grade analyses are provided in S5 Table. Higher grades were associated with an increase in text on all evaluation criteria except for the methods, which received less attention.

Third, we assess the robustness of our results with respect to the linearity assumption of the mixed-effects model. While we keep the gender and disciplinary differences in a linear form, we abandon the linearity assumption for the control variables, which we model fully nonparametrically and learn the functional form of the controls in a data-driven way. In particular, we specify the following partially linear model [4]:

$$Y_{ij} = \alpha + \gamma G_{ij}^{R} + \delta G_{j}^{A} + \theta D_j + f(X_{ij}) + \varepsilon_{ij}$$

where $f(\cdot)$ is an unknown function of the control variables.

To estimate the parameters of the above partially linear models, we follow the recent advances in Double Machine Learning [5]. First, we partial out the coefficients of the policy variables, i.e., gender and discipline, by estimating two nuisance regression models: 1) regressing the outcome on the set of controls and 2) regressing the policy variables on the set of controls. Note that, in practice, we partial out each of the policy variables at a time [6]. We estimate these nuisance regressions fully nonparametrically by Random Forests [7] to allow for any arbitrary functional form. Second, we run a structural residual-on-residual regression via least squares to estimate the coefficients of the policy variables. Such estimated coefficients share the same statistical properties and interpretation as in the linear model. This

is achieved by removing the regularization bias via orthogonalization through partialling out, and by removing the overfitting bias via cross-fitting, i.e., estimating the nuisance regressions and the structural regression on separate sample splits [5]. Within the Double Machine Learning procedure, we also apply clustering on the proposal level [8]. In addition, due to partialling out each of the policy variables at a time, we apply the Bonferroni correction for inference. We present the results of the partially linear models in S6 Table. Estimates were both qualitatively and quantitatively similar to the main results from the linear model, providing evidence that observed differences are not driven by a specific modelling approach (S6 Table).

S4 Table: *Predicting the prevalence of review characteristics by considering interaction effects between the gender of the reviewer and applicant.* Content categories are listed in the first row. Table reports results based on mixed-effects regression models. Coefficients show percentage point differences in prevalence relative to reference category. Models include control variables listed in S2 Table and random intercepts for proposal IDs. 95% confidence intervals in parentheses. Track Record: Applicant's Track Record; Rel., Orig., Topic: Relevance, Originality, Topicality; Methods: Suitability of Methods; Feasibility: Feasibility of Project.

	Evaluation Criteria				Sentiment	
	Track Record	Rel., Orig., Topic.	Methods	Feasibility	Positive	Negative
Reviewer: Female (ref.: Male)	0.87	0.70	0.50	0.50	0.28	-0.63
	[0.56, 1.18]	[0.41, 1.00]	[0.30, 0.70]	[0.33, 0.67]	[-0.25, 0.82]	[-1.03, -0.24]
Applicant: Female (ref.: Male)	0.11	-0.11	0.45	-0.06	0.23	-0.15
	[-0.20, 0.41]	[-0.40, 0.18]	[0.25, 0.66]	[-0.23, 0.11]	[-0.31, 0.77]	[-0.56, 0.25]
Domain: MINT (ref.: LS)	1.81	0.60	-2.19	0.73	4.63	-1.70
	[1.51, 2.10]	[0.33, 0.88]	[-2.39, -1.99]	[0.57, 0.89]	[4.10, 5.15]	[-2.09, -1.30]
Domain: SSH (ref.: LS)	-0.78	0.80	-2.09	-0.45	2.68	1.62
	[-1.10, -0.46]	[0.50, 1.11]	[-2.31, -1.87]	[-0.62, -0.27]	[2.11, 3.25]	[1.19, 2.05]
Reviewer: Female x Applicant: Female	0.42	0.19	-0.07	0.32	1.00	-1.13
	[-0.12, 0.96]	[-0.32, 0.71]	[-0.42, 0.28]	[0.02, 0.62]	[0.06, 1.93]	[-1.83, -0.44]

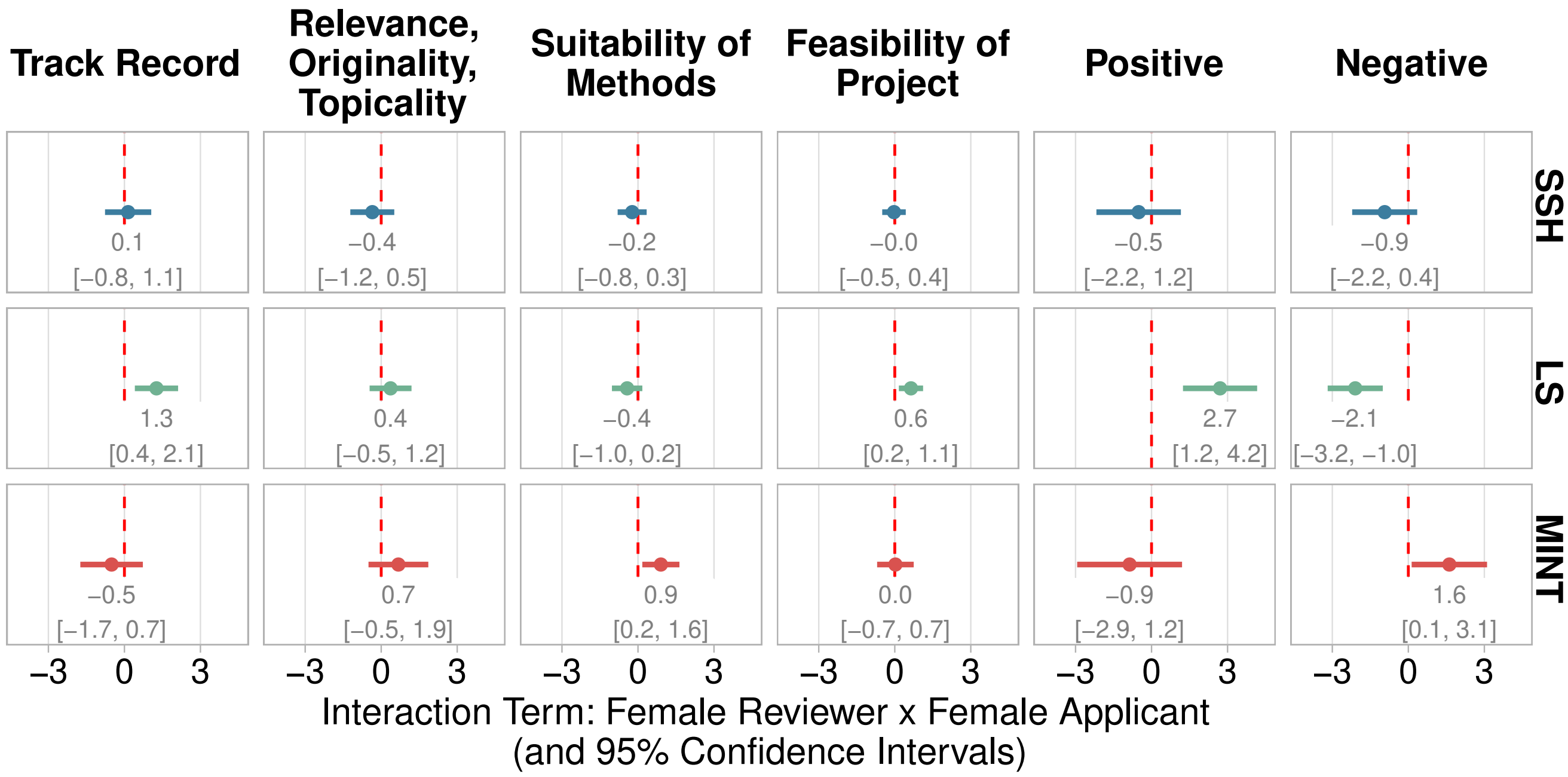

S2 Fig: *Interaction terms for female reviewers and female applicants.* Each box shows the interaction term for a separate model and subset of the dataset for each research area, resulting in 18 regression models. All regression models include the control variables described in the main paper. Horizontal bars depict 95% confidence intervals.

S5 Table: *Predicting the prevalence of review characteristics by the grade assigned by the reviewer.* Content categories are listed in the first row. Table reports results based on mixed-effects regression models. The variable Grade (rescaled) rescales the new grade scale from a nine- to a six-point scale to facilitate comparability across the full sample. Coefficients show percentage point differences in prevalence relative to reference category. Models include control variables listed in S2 Table and random intercepts for proposal IDs. 95% confidence intervals in parentheses. Track Record: Applicant's Track Record; Rel., Orig., Topic: Relevance, Originality, Topicality; Methods: Suitability of Methods; Feasibility: Feasibility of Project.

	Evaluation Criteria				Sentiment	
	Track Record	Rel., Orig., Topic.	Methods	Feasibility	Positive	Negative
Reviewer: Female (ref.: Male)	1.06	0.83	0.48	0.62	0.82	-1.11
	[0.81, 1.31]	[0.59, 1.07]	[0.31, 0.64]	[0.48, 0.76]	[0.46, 1.19]	[-1.37, -0.86]
Applicant: Female (ref.: Male)	0.20	-0.08	0.44	0.02	0.37	-0.33
	[-0.05, 0.45]	[-0.32, 0.16]	[0.26, 0.63]	[-0.12, 0.17]	[0.01, 0.73]	[-0.59, -0.08]
Domain: MINT (ref.: LS)	1.44	0.26	-2.12	0.66	3.18	-0.48
	[1.17, 1.72]	[-0.01, 0.52]	[-2.32, -1.92]	[0.50, 0.81]	[2.78, 3.57]	[-0.76, -0.20]
Domain: SSH (ref.: LS)	-0.67	0.90	-2.10	-0.41	3.01	1.34
	[-0.98, -0.37]	[0.60, 1.19]	[-2.32, -1.89]	[-0.59, -0.24]	[2.58, 3.45]	[1.03, 1.64]
Grade (rescaled from 1-6)	2.49	2.33	-0.45	0.54	9.60	-8.00
	[2.40, 2.58]	[2.24, 2.41]	[-0.51, -0.39]	[0.48, 0.59]	[9.46, 9.73]	[-8.10, -7.91]

S6 Table: *Predicting the prevalence of review characteristics in a partially linear model.* Content categories are listed in the first row. Table reports results based on partially linear regression models estimated via double machine learning with random forest with 1,000 trees as a base learner and 5-fold cross-fitting. Coefficients show percentage point differences in prevalence relative to reference category. Models include control variables listed in S2 Table and clustering on proposal IDs. 95% confidence intervals with Bonferroni correction in parentheses. Track Record: Applicant's Track Record; Rel., Orig., Topic: Relevance, Originality, Topicality; Methods: Suitability of Methods; Feasibility: Feasibility of Project.

	Evaluation Criteria				Sentiment	
	Track Record	Rel., Orig., Topic.	Methods	Feasibility	Positive	Negative
Reviewer: Female (ref.: Male)	0.98	0.86	0.66	0.62	0.83	-0.86
	[0.64; 1.31]	[0.55; 1.18]	[0.44; 0.88]	[0.44; 0.8]	[0.26; 1.4]	[-1.29; -0.43]
Applicant: Female (ref: Male)	0.24	-0.04	0.46	0.01	0.49	-0.35
	[-0.1; 0.58]	[-0.36; 0.28]	[0.23; 0.7]	[-0.17; 0.2]	[-0.09; 1.08]	[-0.79; 0.09]
Domain: MINT (ref.: LS)	1.70	0.46	-2.16	0.65	4.13	-1.66
	[1.31; 2.09]	[0.08; 0.83]	[-2.43; -1.88]	[0.43; 0.87]	[3.45; 4.81]	[-2.18; -1.14]
Domain: SSH (ref.: LS)	-0.70	0.86	-2.16	-0.47	2.31	1.73
	[-1.15; -0.26]	[0.45; 1.28]	[-2.47; -1.84]	[-0.7; -0.24]	[1.52; 3.09]	[1.13; 2.34]

**Full description of SNSF discipline group**	**Short label used in manuscript**
Art Studies; Musicology; Theatre and Film Studies; Architecture	Art and Design
Astronomy; Astrophysics and Space Sciences	Astronomy
Basic Biological Research	Basic Biological Research
Basic Medical Sciences	Basic Medical Sciences
Chemistry	Chemistry
Clinical Medicine	Clinical Medicine
Earth Sciences	Earth Sciences
Economics; Law	Economics and Law
Engineering Sciences	Engineering and Informatics
Environmental Sciences	
Ethnology; Social and Human Geography	Ethnology and Social Geography
Experimental Medicine	Experimental Medicine
General Biology	General Biology
Linguistics and Literature; Philosophy	Linguistics, Literature, and Philosophy
Mathematics	Mathematics
Physics	Physics
Preventive Medicine (Epidemiology/Early Diagnosis/Prevention)	Epidemiology and Preventive Medicine
Psychology; Educational Studies	Psychology and Educational Studies
Social Medicine	Social Medicine
Sociology; Social Work; Political Sciences; Media and Communication Studies; Health	Social and Political Sciences
Theology and Religious Studies; History; Classical Studies; Archaeology	Historical and Religious Studies

S7 Table: *Comparison of 21 SNSF disciplines and the more concise descriptions used in the main text and graphs.* See classification of disciplines here: `https://www.snf.ch/SiteCollectionDocuments/allg_disziplinenliste.pdf`

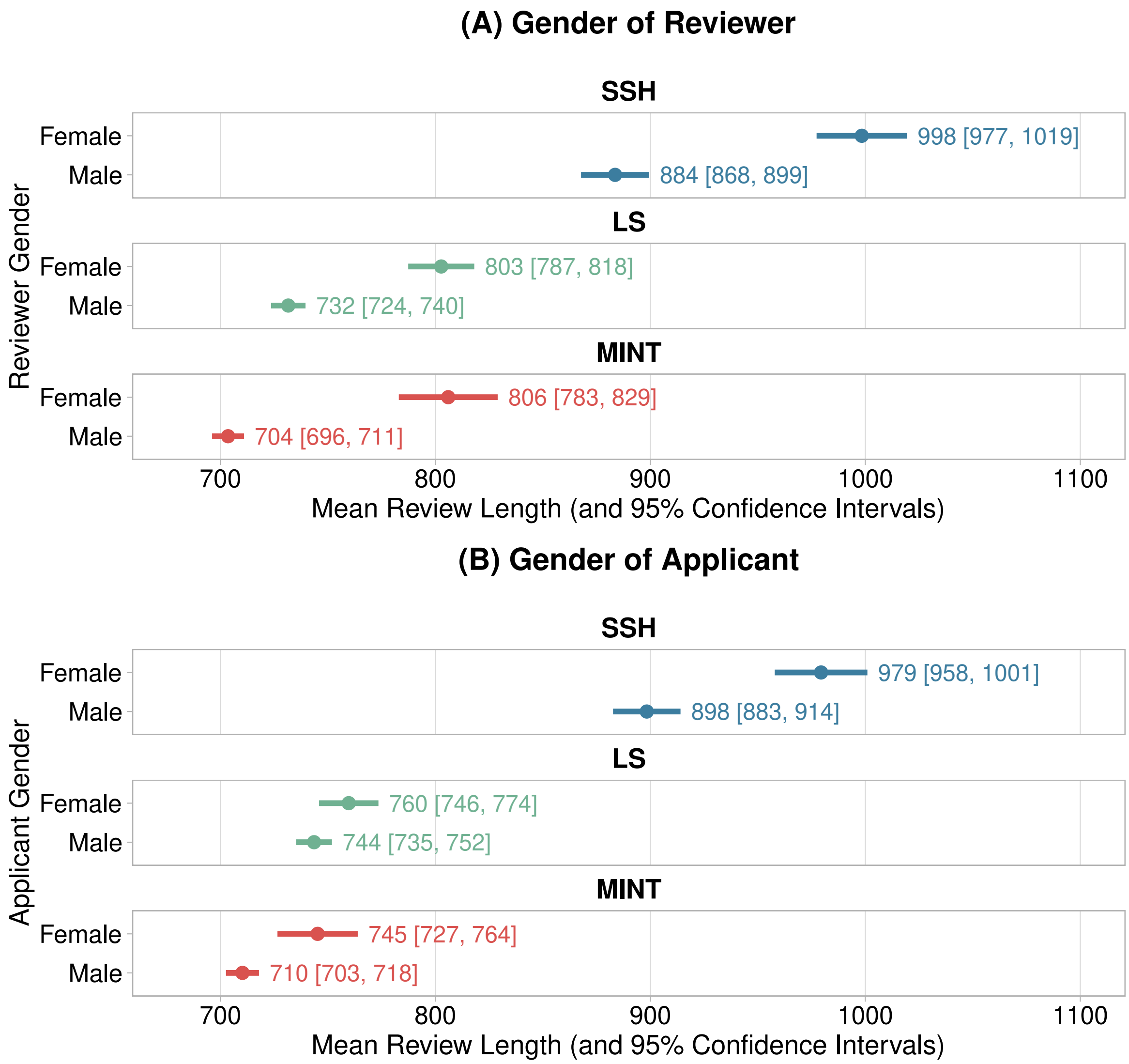

S3 Fig: *Average review length for reports submitted by male and female reviewers (A) and male and female applicants (B).* Dots show mean length (in words), horizontal bars depict 95% confidence intervals. SSH: Social Sciences and Humanities; LS: Life Sciences; MINT: Mathematics, Informatics, Natural Sciences, Technology.

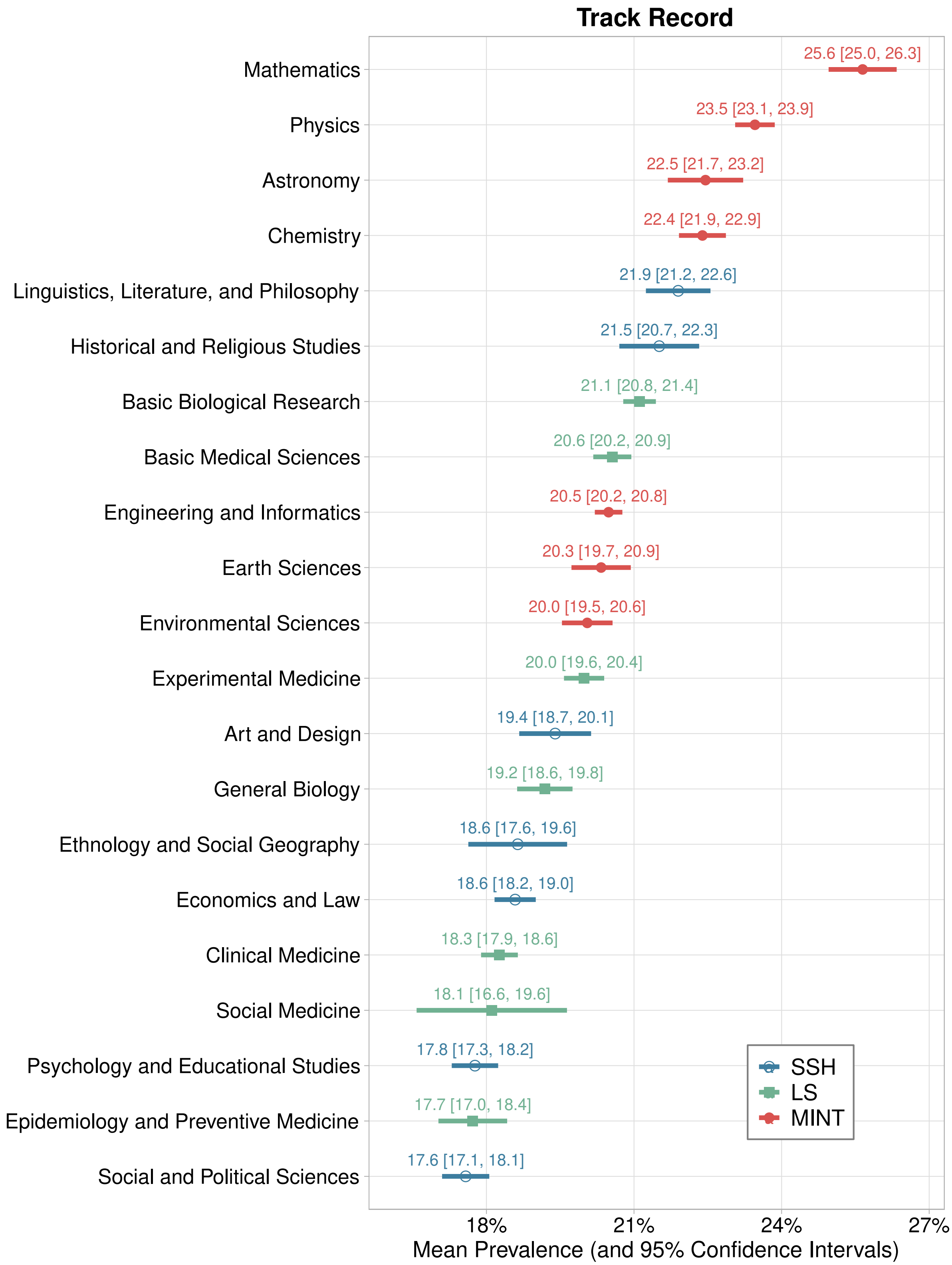

S4 Fig: *Mean prevalence of Track Record (% of review sentences) by discipline.* Horizontal bars depict 95% confidence intervals. Mathematics focuses most on the Applicant's Track Record (25.6%), followed by Physics (23.5%), and Astronomy (22.5%). On the other hand, Social and Political Sciences place the least emphasis on the track record (17.6%).

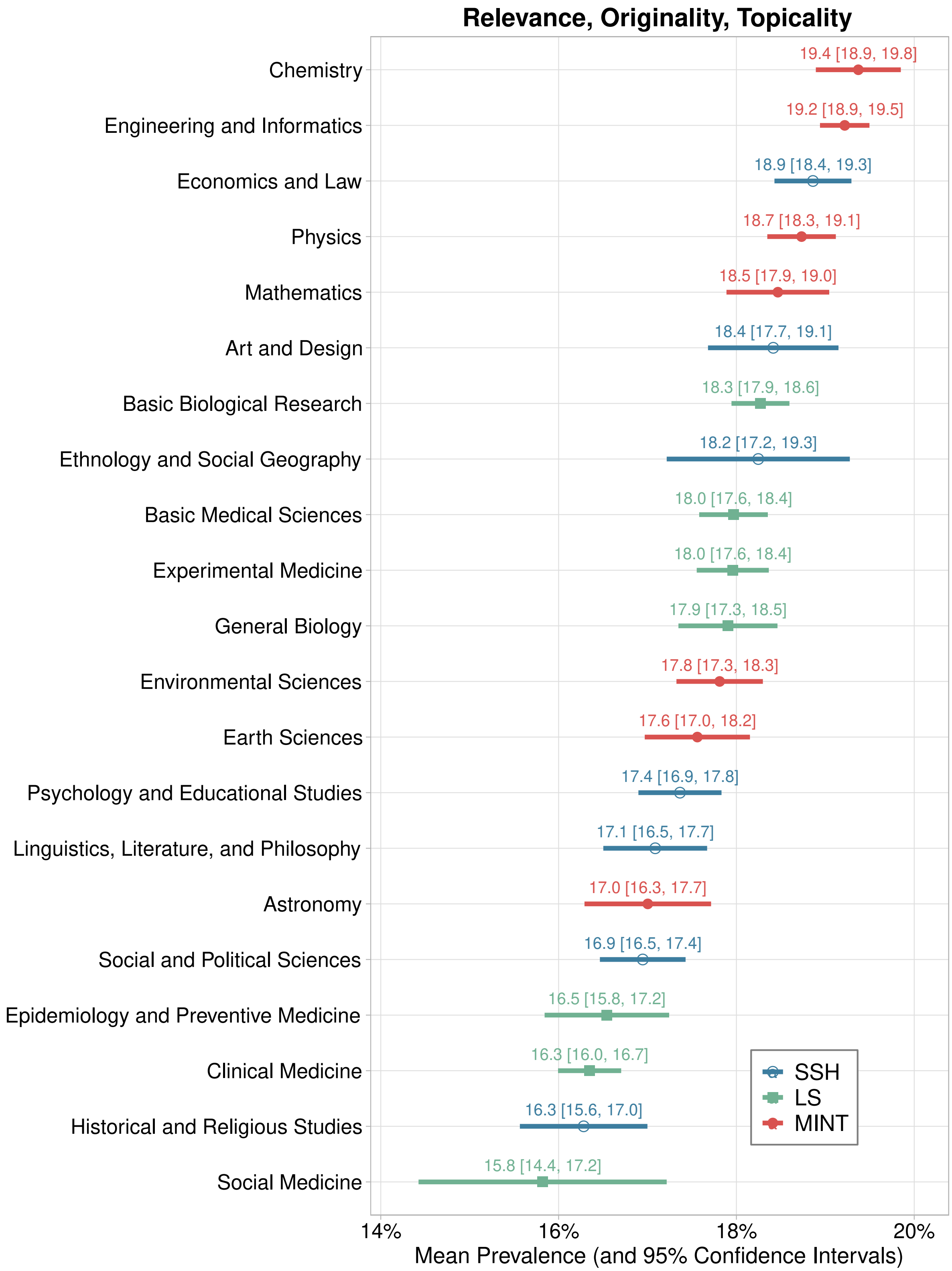

S5 Fig: *Mean prevalence of Relevance, Originality, Topicality (% of review sentences) by discipline.* Horizontal bars depict 95% confidence intervals. Mean differences in the focus on Relevance, Originality, and Topicality tend to be smaller than in other research areas (ranging from 15.8% to 19.4%), and we do not observe clusters across the three research domains.

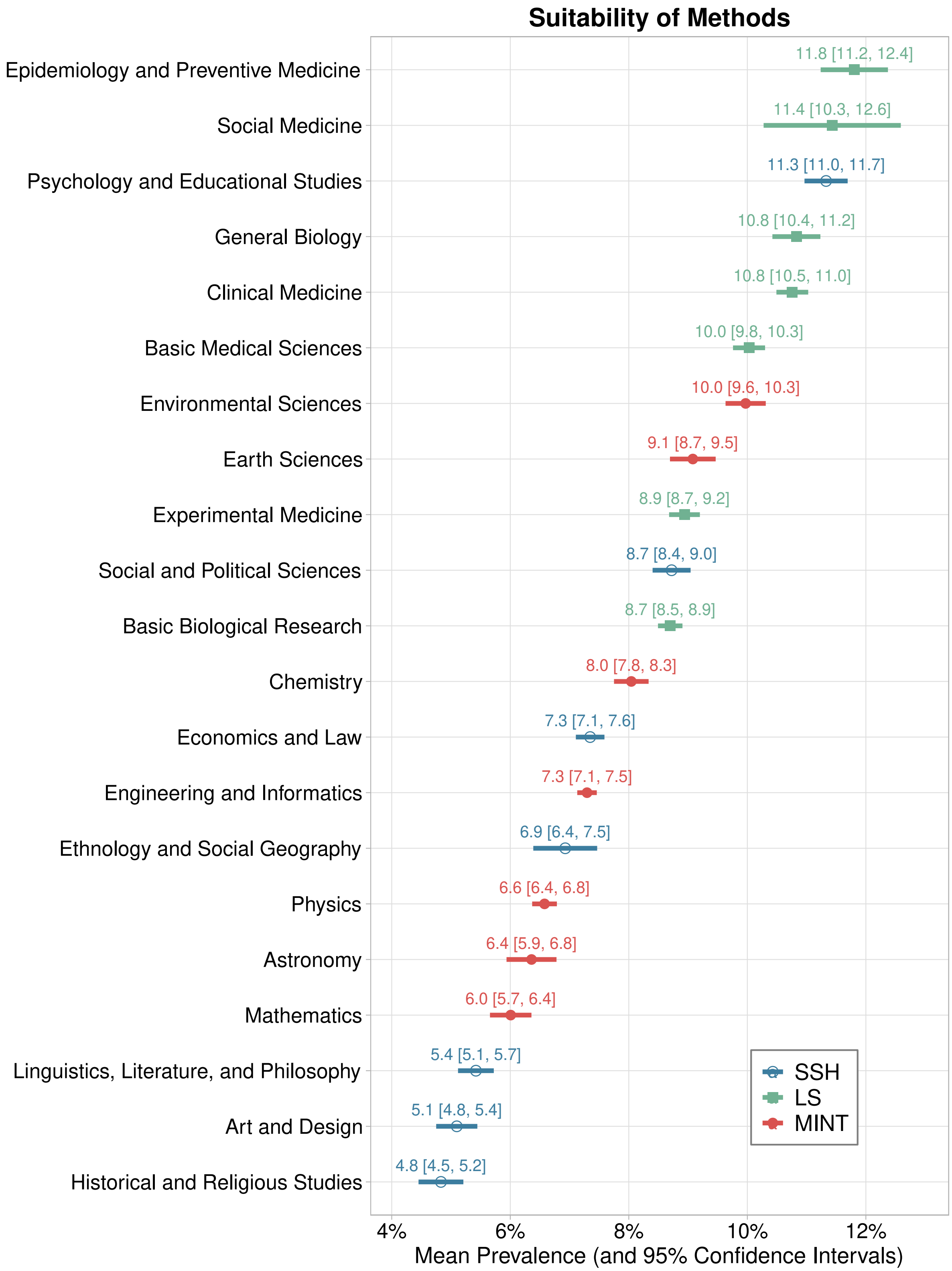

S6 Fig: *Mean prevalence of Suitability of Methods (% of review sentences) by discipline.* Horizontal bars depict 95% confidence intervals. Reviews in the Life Sciences devote considerably more emphasis on the Suitability of Methods (11.8% in Preventive Medicine, 11.4% in Social Medicine) than SSH disciplines, in particular Historical and Religious Studies (4.8%) and Art and Design (5.1%).

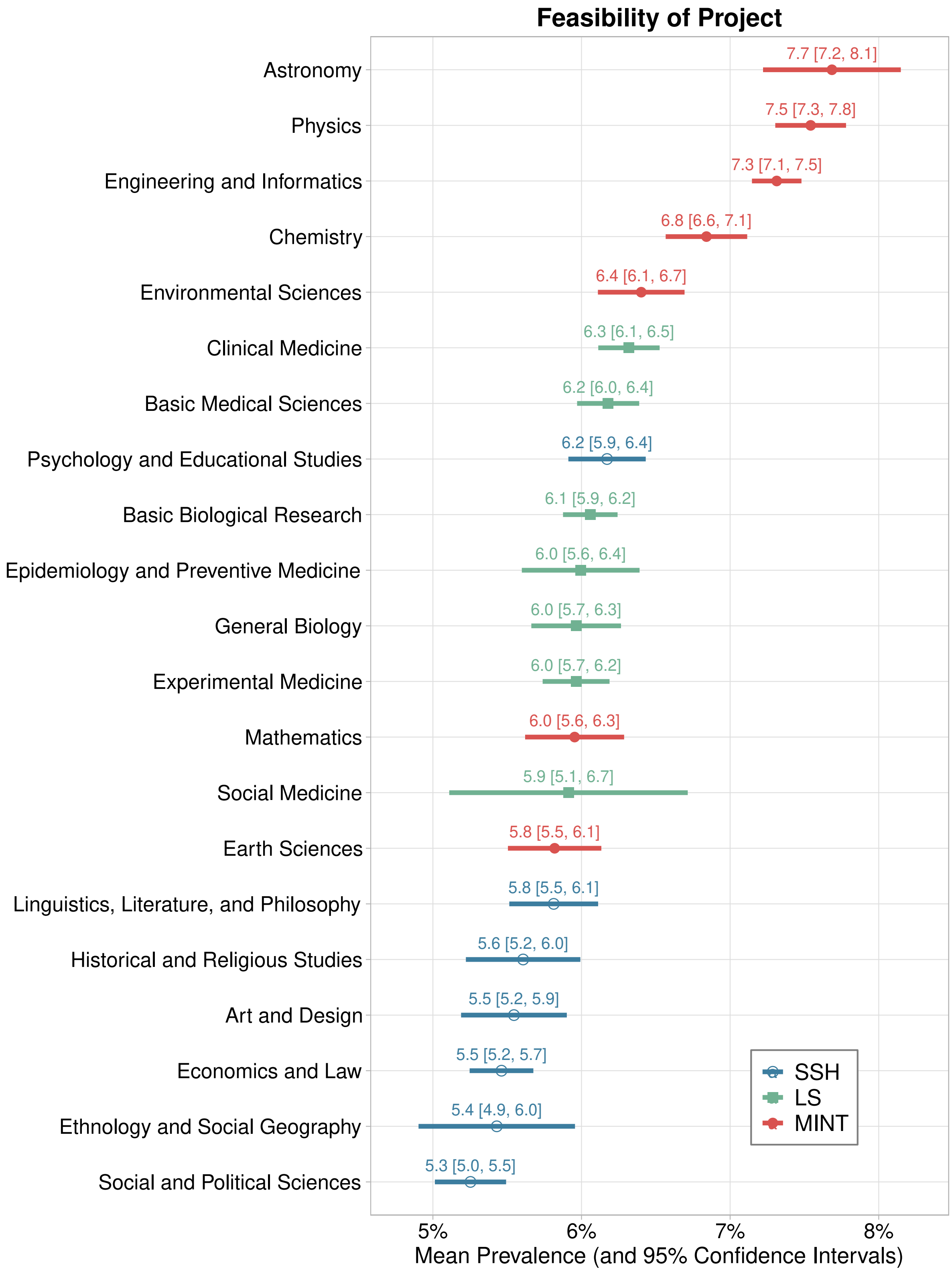

S7 Fig: *Mean prevalence of Feasibility of Project (% of review sentences) by discipline.* Horizontal bars depict 95% confidence intervals. The feasibility of the project is discussed somewhat more extensively in MINT and LS than in SSH, even though these differences are relatively small, ranging from 5.3% in Social and Political Sciences to 7.7% in Astronomy.

S4 Text Data protection, data sharing and responsible use

Language detection, fine-tuning, and sentence-level classifications were conducted locally. The publicly shared data do not contain any identifying information about the authors of the review reports or applicants, and neither the code scripts nor the transformer models allow for the extraction of the raw texts of review reports. All authors and human annotators signed data-sharing agreements, and a Data Management Plan was prepared to ensure compliance [9].

While experiments showed that 2,500 annotated sentences were sufficient to produce reliable classification results for a test set of 500 sentences, the sample is still relatively small. As a result, the classification performance should be carefully evaluated before use, and our models must not be deployed for automatic decision-making without human oversight, as LLMs and Artificial Intelligence (AI) often struggle to predict the quality of research [10] and since these models might reproduce existing biases [11, 12]. Additionally, because the training data is limited to a specific source, the models might not perform well for other types of text or languages other than English.

References

1. Okasa G, de León A, Strinzel M, Jorstad A, Milzow K, Egger M, et al. A Supervised Machine Learning Approach for Assessing Grant Peer Review Reports. Quantitative Science Studies. 2025;6:1189-214. doi:10.1162/qss.a.23.

2. Bondi M, Scott M. Keyness in Texts. John Benjamins Publishing; 2010.

3. Spolsky B. The Length of a Translation. Journal des traducteurs. 1964;9(2):45. doi:10.7202/1061313ar.

4. Robinson PM. Root-N-consistent Semiparametric Regression. Econometrica. 1988;56(4):931. doi:10.2307/1912705.

5. Chernozhukov V, Chetverikov D, Demirer M, Duflo E, Hansen C, Newey W, et al. Double/Debiased Machine Learning for Treatment and Structural Parameters. The Econometrics Journal. 2018;21(1):C1-C68. doi:10.1111/ectj.12097.

6. Bach P, Kurz MS, Chernozhukov V, Spindler M, Klaassen S. DoubleML: An Object-Oriented Implementation of Double Machine Learning in R. Journal of Statistical Software. 2024;108(3). doi:10.18637/jss.v108.i03.

7. Breiman L. Random Forests. Machine Learning. 2001;45(1):5-32. doi:10.1023/A:1010933404324.

8. Chiang HD, Kato K, Ma Y, Sasaki Y. Multiway Cluster Robust Double/Debiased Machine Learning. Journal of Business & Economic Statistics. 2022;40(3):1046-56. doi:10.1080/07350015.2021.1895815.

9. Strinzel M, Okasa G, Jorstad A, Müller S, de León A, Egger M, et al.. Data Management Plan (DMP): A Supervised Machine Learning Approach for Assessing Grant Peer Review Reports; 2024. Available from: `https://doi.org/10.46446/DMP-peer-review-assessment-ML`.

10. Thelwall M, Yaghi A. Evaluating the Predictive Capacity of ChatGPT for Academic Peer Review Outcomes across Multiple Platforms. Scientometrics. 2025;130:5285–5307. doi:10.1007/s11192-025-05287-1.

11. Thelwall M. Research Quality Evaluation by AI in the Era of Large Language Models: Advantages, Disadvantages, and Systemic Effects. arXiv; 2025. doi:10.48550/arXiv.2506.07748.

12. Binz M, Alaniz S, Roskies A, Aczel B, Bergstrom CT, Allen C, et al. How Should the Advancement of Large Language Models Affect the Practice of Science? Proceedings of the National Academy of Sciences. 2025;122(5):e2401227121. doi:10.1073/pnas.2401227121.
