# Supplementary material for: Gender and disciplinary differences in grant proposal peer review: Content and sentiment in 39,280 reports
Source: PLoS One. 2026 Sep 15;21(9):e0352900. doi: 10.1371/journal.pone.0352900 (PMC13577508; doi:10.1371/journal.pone.0352900)
Supplement: Supplementary file 2 [file pone.0352900.s003.pdf]

Authors' response to reviewer comments

Gender and disciplinary differences in grant proposal peer review: Content and sentiment in 39,280 reports

We are grateful to the reviewers for their constructive comments which have greatly helped improve our paper. Below we address these comments and document the changes made to the manuscript.

Reviewer #1

This study examines how gender and disciplinary differences shape grant peer review by analyzing 39,280 reports from the Swiss National Science Foundation (2016–2023). Using supervised machine learning to classify 1.3 million sentences, it finds that SSH reviews are longer and more critical, while MINT reviews are more concise, positive, and track-record-focused compared to LS. Female reviewers write longer, more criteria-aligned, and more positive reports, and female applicants receive slightly more positive sentiments. The results show that gender and disciplinary cultures influence review tone, length, and focus, with implications for fairness in research funding.
Overall, this paper is of interest to the community of peer review mining, text analysis, and so on.
However, this paper has the following weaknesses:

1. The data used in this study are review reports submitted to the Swiss National Science Foundation between 2016 and 2023, which differ from traditional peer reviews of academic papers, such as those available on OpenReview for conference submissions or peer reviews released by certain journals. Therefore, the authors should clearly specify in the title that the study focuses on peer review of research grant proposals. Moreover, the Introduction should provide a more detailed discussion and analysis of the differences between grant peer review and manuscript peer review. Since the study relies solely on SNSF data, the authors should also discuss the representativeness and generalizability of SNSF as a data source.

Response: We appreciate these suggestions and have made various changes.

Our title did explicitly refer to *grant review* but we adjusted the title to clarify that we explicitly focus on peer review of grant proposals. The new title is: "Gender and disciplinary differences in grant proposal peer review: Content and sentiment in 39,280 reports."

We also clarify differences between journal and grant peer review in the introduction section. The paper now states:

> Compared with journal article review, peer review of research grant proposals focuses on the feasibility of a proposed project [6] and requires reviewers to forecast likely outcomes rather than evaluate completed work [7]. Grant review also typically follows a single-round process with no opportunity for revision, whereas journal peer review usually involves one or more rounds of revision and resubmission.

We also discuss the representativeness and generalizability of SNSF grant reviews as a data source in the last paragraph of section "Characteristics of peer reviewers and applicants":

> Although the dataset was drawn exclusively from the SNSF, it spanned a wide range of scientific disciplines, included reviewers from many countries, and showed broadly proportional gender distributions among both reviewers and applicants. Together, these features support the relevance of our findings beyond a single disciplinary or national setting. In addition, the SNSF's evaluation criteria align with widely used international research assessment practices, including commitments associated with the San Francisco Declaration on Research Assessment (DORA) [30] and the adoption of narrative CV formats [31].

2. The study uses Google's Compact Language Detector 2 for language identification. The authors should provide necessary information about the accuracy and reliability of this tool.

Response: We appreciate this comment, since the accurate identification of languages is crucial for subsequent analyses. We used Google's Compact Language Detector 2 to identify English-language reviews. CLD2 internally relies on a Naïve Bayes classifier. CLD2 is designed for texts of at least 200 characters (approximately two sentences) and is not optimised for very short texts. We therefore detect languages based on the entire text of a reviewer's response to a question (each review is split into several text boxes). This approach ensures higher classification accuracy than a sentence-level approach. Afterwards, we keep only sentences stemming from texts classified as English.

To empirically validate this approach, we manually assessed a random sample of 1,000 sentences from the full dataset during the revision process. One of the authors read each sentence and assessed whether the text was English or written in another language. We found that 991 of 1,000 sentences (99.1%) were correctly classified as English. The nine misclassifications can be categorised as follows: three cases involved references in other languages (one English reference embedded in non-English text, one German reference, and one standalone reference), one case represented a genuine error (a German reference within English text), and six cases stemmed from HTML code or coding issues. These errors largely arise from formatting artefacts rather than genuine language detection failures, suggesting that CLD2 performs reliably for the purpose of this study.

We added the following text to the manuscript:

> We restricted our analysis to English-language reviews using Google's Compact Language Detector 2 (CLD2) [28]. This model builds on a computationally efficient Na¨ıve Bayes classifier. Because CLD2 was optimized for texts of at least 200 characters (approximately two sentences), we applied language detection to the full text of each reviewer's response to an evaluation criterion rather than at the sentence level. We then retained only sentences from texts classified as English. To empirically validate this approach, we manually reviewed 1,000 randomly sampled sentences: 99.1% of sentences were assigned to the correct language. The nine misclassifications comprised two cases involving literature references in other languages, one genuine language detection error, and six instances of special characters due to formatting

issues. The validation demonstrates the accuracy of the approach, confirming previous studies [29].

3. The study adopts traditional five-fold cross-validation. To better avoid potential overfitting, the authors should explain why the dataset was not split into a training set, validation set, and test set.

Response: Thank you for this suggestion. Within the fine-tuning process, we did indeed split the dataset into training, validation, and test set. We *additionally* performed the training and validation split five times within a cross-validation schema to obtain a more robust estimate of the validation error that is not sensitive to one particular data split as suggested e.g. by (Hastie, Tibshirani, and Friedman 2009). This was important as we have faced class imbalances in our dataset, which would make the evaluation more prone to specific data split dependency.

Apart from the cross-validation schema, we have additionally evaluated the classification accuracy based on an untouched test set as well. We have rewritten the paragraph and explained the fine-tuning procedure in more detail:

> We fine-tuned six separate transformer machine learning models for a binary text classification task, one for each content category. We evaluated classification accuracy by splitting the 3,000 annotated sentences into training (2,000), validation (500), and test (500) sets, repeating the train-validation split five times within a cross-validation schema to obtain a more robust estimate of validation error that is less sensitive to any single split [36]. The final models were then fine-tuned using the full set of 2,500 sentences and evaluated on the untouched test set of 500 sentences. Among multiple tested pre-trained transformer models, fine-tuning the SPECTER2 model [37,38] for each category yielded the best performance as measured by the macro-averaged F1-score, both regarding cross-validation as well as test error.

4. The authors should provide a necessary explanation of the basic principles underlying keyness analysis.

Response: We have added a new section to the Appendix (S1 Text: Keyness analysis methodology) explaining the chi-squared approach to keyness analysis, including the formula, a worked example, and implementation details. This addition clarifies how we identified the most characteristic terms for each content category reported in Table 2.

The main text now includes:

> To identify the most characteristic terms for each content category, we conducted keyness analyses using χ 2 tests, comparing word frequencies in sentences where a given characteristic was present (target group) with those where it was absent (reference group) [21,46–48]. For each category, we ranked all words by their χ 2

values and report the top 25 terms in Table 2. Full details of the approach, including the formula and a worked example, are provided in S1 Text.

S1 Text includes the following content:

We conducted so-called keyness analyses using chi-squared (χ^2) tests to identify the most characteristic terms for each content category. We performed separate analyses for each of the six content categories (Track Record; Relevance, Originality, Topicality; Suitability of Methods; Feasibility of Project; Positive; Negative). For each category, we split the corpus into sentences where the characteristic was present (target group) and sentences where it was absent (reference group). Since sentences could be assigned to multiple categories, the same sentence might appear in the target group for one category and the reference group for another.

Within each analysis, we calculated the chi-squared statistic for every word and previously identified multi-word expression, comparing its frequency in the target group with its frequency in the reference group. The chi-squared statistic measures how much observed word frequencies deviate from expected frequencies under independence. The formula is:

$$\chi^2 = \frac{N \times (ad - bc)^2}{(a+b)(c+d)(a+c)(b+d)}$$

where *a* is the frequency of the word in the target group, *b* is its frequency in the reference group, *c* is the frequency of all other words in the target group, *d* is the frequency of all other words in the reference group, and *N* is the total word frequency across both groups. Higher χ^2 values indicate stronger association between a word and the target category. For each category, we ranked all words by their χ^2 values.

To illustrate the approach, we provide a simple example. Consider the word 'methodology' when comparing sentences about the Suitability of Methods (n = 10,000 words, including 'methodology' 50 times) with other sentences (n = 90,000 words, including 'methodology' 30 times). The chi-squared statistic is:

$$\chi^2 = \frac{100{,}000 \times (50 \times 89{,}970 - 30 \times 9{,}950)^2}{(80)(99{,}920)(10{,}000)(90{,}000)} = 245.20$$

Keyness values were calculated using the *textstat_keyness()* function from the *quanteda.textstats* package (version 0.97) in R (version 4.4.2), with *measure = "chi2"*. This implementation does not apply Yates's continuity correction, which is standard practice in corpus linguistics for large samples [2].

5. The description of the annotation process needs additional detail, including the annotators' professional backgrounds, how annotation quality was ensured, and the results of inter-annotator agreement tests.

Response: We thank the reviewer for this comment. Detailed information on the annotation process is provided in our companion paper (Okasa et al., 2025, pp. 1192–1194), which we now reference more clearly in the Methods section.

> Four annotators participated in the study. All had academic backgrounds and experience with peer review, and the team included both native and non-native English speakers, reflecting the international pool of reviewers who submit reports to the SNSF.
>
> Annotators were trained to recognize that evaluation criteria may be expressed differently across research domains; for example, methodological discussions can differ substantially between the humanities and the natural sciences. They were instructed to assign labels based on conceptual meaning rather than specific terminology, thereby accounting for disciplinary variation in evaluative language.
>
> We implemented several measures to ensure high-quality annotations. The annotation codebook was developed through multiple rounds of piloting and refinement. To reduce individual bias, each sentence was independently labeled by three annotators, and final classifications were determined by majority vote. After each annotation round, we systematically analyzed patterns of agreement and disagreement and used these insights to iteratively improve the guidelines. We also calculated full agreement rates (i.e., cases where all three annotators assigned identical labels) for each category. Across the six categories used in this paper, full agreement ranged from 74% to 90% (mean: 83%).

6. The classification model used in this study is relatively limited. The authors could introduce additional state-of-the-art models to compare model performance and stability.

Response: We appreciate this suggestion. In our companion paper, focused on the methodological aspects, we compared fine-tuning SPECTER2 with two alternative transformer models: the baseline BERT and RoBERTa (a robustly optimised version of BERT) as well as with few-shot learning approach using Llama 3 8B model. We extensively validate these classifiers in (Okasa, de León, et al. 2025), where the SPECTER2 model achieved the best performance. We added the following paragraphs to the manuscript:

> We opted for fine-tuning a SPECTER2 transformer model for several reasons. First, it was pre-trained on scientific texts, making it well-suited to peer review reports [35,39–41]. Second, it supports full transparency and reproducibility: we release our fine-tuned models on Hugging Face (https://huggingface.co/snsf-data), enabling other funding agencies to apply them to their own data. Third, in our comparisons SPECTER2 outperformed a few-shot approach using a large language model (Llama 3 8B [42]), especially for imbalanced categories [32], consistent with evidence that fine-tuned smaller models could match or exceed zero-shot LLM approaches on targeted classification tasks [43]. Finally, SPECTER2 is computationally efficient and can be run locally, avoiding reliance on proprietary services and reducing privacy risks when analyzing confidential review texts.

> Overall, our approach prioritizes performance and practical utility. BERT-based models are well established in peer review text analysis [21,44], and our broader model comparison shows that fine-tuned transformers offer a strong balance of accuracy, reproducibility, and computational efficiency [32].

7. The study does not include institutional prestige or related latent variables. Incorporating such factors as control variables may improve the explanatory power and robustness of the models.

Response: Thank you. We agree that institutional prestige and related latent factors can influence review content and that accounting for them can strengthen model robustness. While we could not include a direct prestige measure, our models do include applicants' institutional affiliations, grouped by institution type (ETH domain, cantonal universities, universities of applied sciences/teacher education, university hospitals, and other research institutions). In the Swiss context, these categories are correlated with institutional prestige and resources. As with any observational analysis, residual confounding by unobserved factors remains possible. We now clarify in the Discussion that our estimates should be interpreted as conditional associations and we explicitly note the limitation that we cannot fully rule out unmeasured prestige-related influences beyond the affiliation proxy:

> The set of potential confounding variables was not exhaustive, which may limit model robustness and explanatory power. For example, we could not directly assess institutional prestige but included applicants' institution type as a proxy. Because the study is observational, our estimates should be interpreted as conditional associations rather than causal effects. Nevertheless, we adjusted for a range of observable characteristics that could influence both review content and our variables of interest, i.e., reviewer and applicant gender, and proposal discipline. Further, gender was treated as binary and assigned only to the corresponding applicant, potentially oversimplifying the diversity of applicant and team identities. Finally, sentiment analysis may miss subtle or discipline-specific forms of critique or praise [60].

8. It is recommended that the Discussion section be expanded appropriately, with a deeper analysis of both the theoretical implications and the practical implications of the study's findings.

Response: Thank you for this suggestion. We agree that the paper would gain from a more in-depth discussion of theoretical implications and practical implications. We have expanded the discussion section as follows:

> Our study helps move peer review research beyond describing "deficits" (e.g., low agreement or bias) toward the mechanisms that produce observed patterns. Recent conceptual work argues that peer review is best understood as a set of interrelated practices that generates (i) expert judgments of quality, (ii) allocation decisions under scarcity, and (iii) legitimacy for these decisions within science and to external audiences [56,57]. The disciplinary differences in how criticism and praise are

expressed suggest that peer review reflects field-specific evaluative cultures rather than functioning as a uniform measurement instrument: what counts as a "quality signal" may vary across disciplines. Status- and role-based theories further predict that gender can shape competence expectations and standards for evaluating ability [58,59] , which may translate into systematic differences in how reviewers describe applicants and frame strengths and weaknesses.

Limitations include the inability to assess how gender and disciplinary differences affected panel discussions or funding outcomes, the restriction to English-language reports, and the observational design, which limits causal inference.

External peer review at the SNSF is only one stage in a multi-step evaluation process. Funding decisions are made during panel deliberations, where external reviews are discussed, interpreted, and weighed alongside internal assessments, but these discussions are not available for systematic analysis. We show that review characteristics vary by gender and disciplinary context, yet we cannot assess whether these differences translate into funding disparities. Addressing this question would require research designs that capture panel-level decision-making, which is beyond the scope of this study. In addition, because non-English reports were excluded, our findings apply only to English-language peer review reports. This includes virtually all reports in LS and MINT, but only about two-thirds of reports in SSH. The results should therefore be interpreted with particular caution for SSH, where inclusion of non-English reports might have changed the observed patterns.

The set of potential confounding variables was not exhaustive, which may limit model robustness and explanatory power. For example, we could not directly assess institutional prestige but included applicants' institution type as a proxy. Because the study is observational, our estimates should be interpreted as conditional associations rather than causal effects. Nevertheless, we adjusted for a range of observable characteristics that could influence both review content and our variables of interest, i.e., reviewer and applicant gender, and proposal discipline. Further, gender was treated as binary and assigned only to the corresponding applicant, potentially oversimplifying the diversity of applicant and team identities. Finally, sentiment analysis may miss subtle or discipline-specific forms of critique or praise [60].

While 2,500 annotated sentences were enough to achieve reliable performance on a 500-sentence test set, the sample remains small, so classifications should be used cautiously. The classifiers should not be used for automated decision-making without human oversight, given the known limitations of large language models, including difficulties in assessing research quality and risks of reproducing biases. Further, our classification models may underestimate rare but meaningful forms of evaluative language specific to particular disciplines. Discipline-specific rhetoric that diverges substantially from the training data may therefore be misclassified. S4 Text provides further details on data protection, data sharing and responsible use.

Our results have practical implications: if disciplines differ in how they express criticism, confidence, or praise, reports may not be directly comparable across fields. This strengthens the case for structured review templates and well-defined criteria that prompt reviewers to address the same dimensions consistently and separate criterion-based judgments from overall sentiment. Mandatory justification fields could further

increase transparency and help panels interpret reviews. The evidence on training for grant reviewers remains limited [61]. A randomized trial of automated feedback found little improvement in score agreement [62], indicating that training should focus on how to interpret criteria rather than on scoring patterns. Panel-level calibration may benefit from brief pre-panel discussions of sample applications and benchmarks, supported by written guidance. Finally, large language models could support quality assurance by analyzing review texts over time; for example, monitoring whether some panels provide systematically more negative or less criterion-specific feedback, or whether interventions reduce variation.

Reviewer #2

I have reviewed the paper entitled "Gender and disciplinary differences in grant review: Analysis of content and sentiment of 39,280 peer review reports". The topic of the paper is interesting and relevant. However, it has some caveats that need to be addressed before it is ready to be published.

Response: We thank the reviewer for the positive assessment of our manuscript and the helpful improvement suggestions.

1. My first comment is that the analysis excludes non-English peer review reports, despite the SNSF allows reviews in German, French, and Italian, particularly in SSH disciplines. This restriction may introduce a selection bias, as linguistic choice could correlate with discipline, reviewer seniority, or cultural norms in evaluation. The manuscript acknowledges this limitation briefly, but does not assess whether excluded reviews differ systematically in length, tone, or content. A sensitivity analysis or descriptive comparison would strengthen confidence in the generalizability of the findings.

Response: We thank the reviewer for this important point. Excluding non-English reviews may introduce selection bias because language is not randomly distributed across disciplines: virtually all reports in LS and MINT were written in English, whereas in SSH about one-third were written in German (21.9%), French (9.4%), or Italian (0.4%).

We therefore agree that our findings should be interpreted as applying to English-language SNSF reviews. A full sensitivity analysis was not feasible because our annotation and transformer-based classification pipeline was developed and validated only for English-language texts. We analyzed the length of peer review reports – see new S1 Fig. in the appendix, reproduced below.

The figure shows that review length varies considerably across the four languages in the corpus.

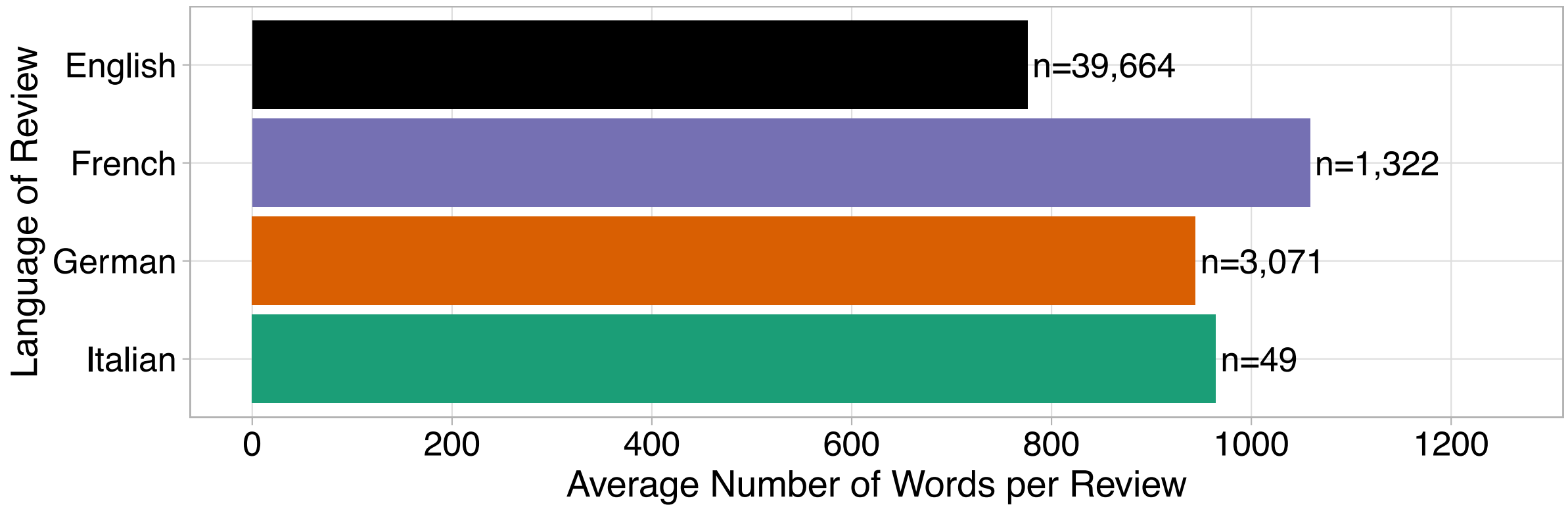

S1 Fig: Comparison of average review length across languages.

However, the interpretation of these differences in length is difficult. It will be partly due to the in general longer review texts in the SSH, but also due to the well-known fact that English texts tend to be shorter than German, French or Italian translations (Spolsky 1964).

To make the language-related limitation of our study more transparent, we now report the language distribution across disciplines and clarify this point in the Discussion.

> Limitations include the inability to assess how gender and disciplinary differences affected panel discussions or funding outcomes, **the restriction to English-language reports**, and the observational design, which limits causal inference. External peer review at the SNSF is only one stage in a multi-step evaluation process. Funding decisions are made during panel deliberations, where external reviews are discussed, interpreted, and weighed alongside internal assessments, but these discussions are not available for systematic analysis. We show that review characteristics vary by gender and disciplinary context, yet we cannot assess whether these differences translate into funding disparities. Addressing this question would require research designs that capture panel-level decision-making, which is beyond the scope of this study. **In addition, because non-English reports were excluded, our findings apply only to English-language peer review reports. This includes virtually all reports in LS and MINT, but only about two-thirds of reports in SSH. The results should therefore be interpreted with particular caution for SSH, where inclusion of non-English reports might have changed the observed patterns.**

2. It is not clear if only 3,000 sentences were manually annotated out of more than 1.3 million sentences.

Response: The reviewer is correct that we annotated 3,000 sentences (evaluations by three annotators for each of these sentences) from a corpus of over 1.3 million sentences. This sample size may seem small relative to the full corpus, but an ablation study (Okasa, De León, et al. 2025) 1206–1207 and Figure 9) demonstrates that additional training data beyond approximately 2,000–2,500 sentences yielded only marginal improvements in classification accuracy. For most categories, particularly those with balanced class distributions, high classification accuracy was achieved even with smaller samples. This is typical for fine-tuned transformer models, which can achieve strong performance with relatively modest amounts

of task-specific training data (Tunstall et al. 2022) as documented in related empirical studies (Forster et al. 2024; Ghosal et al. 2022; Heidler, Rohden, and Funk 2025). Given this evidence and the substantial cost of obtaining additional annotated data, we determined that 2,500 sentences in the training set (with 500 held out for testing) provided an optimal balance between annotation effort and model performance. We have added the following paragraph to clarify this point:

> The annotated dataset of 3,000 sentences constituted a representative sample from a corpus of over 1.3 million sentences, while providing a sufficient amount of training data for fine-tuning the transformer models. An ablation study showed that additional training data beyond 2,000 sentences yielded only marginal improvements in classification accuracy [32]. For most categories, particularly those with balanced class distributions, high classification accuracy was achieved even with smaller samples. This is typical for fine-tuning transformer models, which can achieve strong performance with relatively modest amounts of task-specific training data [33], as also documented in related empirical studies [17,34,35].

3. The paper does not sufficiently discuss potential domain imbalance in the annotated sample (e.g., SSH vs. MINT) or whether rare but meaningful forms of evaluative language are underrepresented. My concern is related to specific rhetoric of disciplines that could be misclassified or missed.

Response: We appreciate this concern and have clarified two key points:

Domain representativeness: The annotated sample closely mirrors the full corpus distribution: 38.3% MINT, 36.9% LS, and 24.7% SSH (Okasa, De León, et al., 2025, p. 1192), ensuring no systematic over- or underrepresentation of research domains.

Discipline-specific rhetoric: Annotators were explicitly trained to recognise that evaluation criteria may be discussed differently across research domains (e.g., methodological discussions vary between humanities and natural sciences). They were instructed to apply labels based on conceptual meaning rather than specific terminology (Okasa, De León, et al., 2025, p. 1193).

We now explicitly acknowledge in the Limitations section of the Discussion that our models may underrepresent rare discipline-specific evaluative language.

> Annotators were trained to recognize that evaluation criteria may be expressed differently across research domains; for example, methodological discussions can differ substantially between the humanities and the natural sciences. They were instructed to assign labels based on conceptual meaning rather than specific terminology, thereby accounting for disciplinary variation in evaluative language.
>
> Further, our classification models may underestimate rare but meaningful forms of evaluative language specific to particular disciplines. Discipline-specific rhetoric that diverges substantially from the training data may therefore be misclassified.

4. The study is explicitly observational, yet some interpretations seems to imply causality. For example: gender or disciplinary culture "shaping" review content. While regression controls are extensive, unobserved confounders such as institutional prestige, prior collaboration networks, or proposal quality could have an effect in reviewer's opinion.

Response: Thank you for the comment. We acknowledge the observational nature of our analysis and the possibility of not capturing all potential confounding variables, especially the unobserved latent factors. To address these concerns explicitly, we have added the following paragraph to the manuscript mentioning the limitations in causal identification (see also comment #7 by Reviewer 1):

> The set of potential confounding variables was not exhaustive, which may limit model robustness and explanatory power. For example, we could not directly assess institutional prestige but included applicants' institution type as a proxy. Because the study is observational, our estimates should be interpreted as conditional associations rather than causal effects. Nevertheless, we adjusted for a range of observable characteristics that could influence both review content and our variables of interest, i.e., reviewer and applicant gender, and proposal discipline. Further, gender was treated as binary and assigned only to the corresponding applicant, potentially oversimplifying the diversity of applicant and team identities. Finally, sentiment analysis may miss subtle or discipline-specific forms of critique or praise [60].

In our view, linear causal thinking is not appropriate in this context. Review language is best understood as the result of several interacting institutional, disciplinary, social, and evaluative processes rather than simple one-way causation. In this context, discipline, gender, quality judgments, and review language are not fully separate factors with clear one-directional effects but closely connected parts of the evaluation process.

Nevertheless, we have removed "causal language" from the Results section and toned down causal interpretation. We have reworded the following sentences:

Abstract

Original: "Gender and disciplinary culture influence how grant proposals are reviewed, shaping the tone, length, and focus of peer review reports"

Changed to: "The tone, length, and focus of peer review reports **varied systematically** by applicant gender and disciplinary context."

Results Section

Original: "positive comments increased substantially with higher grades"

Changed to: "positive comments were strongly associated with higher grades"

Original: "negative comments decreased"

Changed to: "reviews with higher grades contained fewer negative comments"

Conclusions

Original: “disciplinary differences and reviewer gender affect the tone and content”
Alternative: “disciplinary differences and reviewer gender relate to the tone and content of grant peer review reports”

5. Although the paper convincingly shows variation in review content and sentiment, it does not assess whether these differences translate into funding decisions. This is important to know if observed gender or disciplinary differences have consequences in resources. A structural limitation is that funding decisions are often shaped by panel deliberations where reviews are interpreted, weighted, or overridden. But the paper does not include a panel level analysis of the complete evaluation process.

Response: We thank the reviewer for this important observation about the link between review content and funding outcomes. We agree that this is a key question for understanding whether differences in review practices affect resource allocation. The SNSF evaluation procedure involves several sequential stages: external review, internal review, panel discussion and rating, funding decisions, and communication of results. Our study examines only the external review stage.

Funding decisions result from a more complex process in which evaluation panels discuss the external reviews, conduct their own assessments, and reach collective decisions through deliberation, voting, proposal ranking, and the setting of a funding line (Heyard et al. 2022). During these deliberations, external reviews may be interpreted, weighted, and recontextualized. Therefore, even if we observe systematic differences in review content or sentiment across disciplines or reviewer gender, we cannot determine how these differences translate into final funding outcomes without data on the full deliberative process.

Our findings show that gender and disciplinary context are associated with differences in written reviews. Determining whether these differences lead to funding disparities would require a different research design that captures panel-level dynamics and decision-making processes. This remains an important direction for future research, as highlighted in the last sentence of the Conclusions.

We have clarified this limitation in the Discussion section to help readers understand the broader evaluation context within which external reviews operate.

> External peer review at the SNSF is only one stage in a multi-step evaluation process. Funding decisions are made during panel deliberations, where external reviews are discussed, interpreted, and weighed alongside internal assessments, but these discussions are not available for systematic analysis. We show that review characteristics vary by gender and disciplinary context, yet we cannot assess whether these differences translate into funding disparities. Addressing this question would require research designs that capture panel-level decision-making, which is beyond the scope of this study. In addition, because non-English reports were excluded, our findings apply only to English-language peer review reports. This includes virtually all reports in LS and MINT, but only about two-thirds of reports in SSH. The results should therefore be interpreted with particular caution for SSH, where inclusion of non-English reports might have changed the observed patterns.

Hope you find my comments useful to improve your paper.

Response: These suggestions were very helpful and certainly improved the manuscript.
