# Supplementary material for: Gender and disciplinary differences in grant proposal peer review: Content and sentiment in 39,280 reports
Source: PLoS One. 2026 Sep 15;21(9):e0352900. doi: 10.1371/journal.pone.0352900 (PMC13577508; doi:10.1371/journal.pone.0352900)
Supplement: Supplementary file 3 [file pone.0352900.s004.pdf]

Authors' response to reviewer comments (round 2)

Gender and disciplinary differences in grant proposal peer review: Content and sentiment in 39,280 reports

We are grateful to the reviewers for their further constructive comments on our revised submission. Below we address these comments and document the changes made to the manuscript.

Reviewer #1 (Chengzhi Zhang): It is recommended that the description of the SNSF data from 2016 to 2023 in the last paragraph of the Introduction be moved to the Materials and Methods section to improve the readability of the data source description.

Authors' response: Thank you for this comment. We have revised the last paragraph of the Introduction to read:

The Swiss National Science Foundation (SNSF) supports basic research and use-inspired basic research across all disciplines. Its largest funding scheme, Project Funding, supports investigators pursuing self-chosen topics. Project proposals are reviewed by at least two external experts, who evaluate the applicant's track record, the research's relevance and originality, the suitability and feasibility of the methods, and assign an overall score. We compared content and sentiment of peer review reports used to evaluate SNSF project grant proposals.

The first sentence of the first paragraph of the Materials and methods section was revised as follows:

The full sample of peer review reports contained 44,026 reports written in English, German, French, or Italian, pertaining to 11,981 proposals submitted between October 2016 and April 2023 across 21 disciplines in the Social Sciences and Humanities (SSH), Life Sciences (LS), and Mathematics, Informatics, Natural Sciences, and Technology (MINT).

Reviewer #2: The authors have made a serious effort to address the reviewer's concerns, particularly regarding causal interpretation and model limitations. However, some key issues – especially the potential selection bias introduced by excluding non-English reviews – are acknowledged but not fully resolved through additional empirical analysis. As such, while the manuscript has improved, certain methodological concerns remain only partially addressed.

Authors' response: We aimed to examine how the content and tone of grant-proposal peer-review reports varied by discipline, reviewer gender, and applicant gender. Most reports were written in English (90%); we therefore developed an English-language large language model.

We found that reviewers' language in the 39,280 reports varied systematically by field and gender. SSH reviews were longer and more critical; MINT reviews were shorter, more positive, and more focused on applicants' track records; female reviewers wrote longer, more criterion-aligned, and somewhat more positive reviews; and female applicants received slightly more positive and less negative comments.

The aim of our study was not to investigate the use of languages other than English in SNSF peer-review reports. Nevertheless, in the revised manuscript we gave this issue greater attention by validating the language detector and making the language restriction more transparent in the Methods. In the Discussion, we clarified that our findings apply only to English-language peer-review reports.

We have now further revised paper, updating the description of the full sample and subsets and adding an additional supplementary table (S3 Table) to illustrate this point. We also extended S2 Text and updated S1 Fig.

S3 Table, copied below, shows that English-language reviews dominated overall. In Life Sciences and MINT, almost all reviews were in English whereas in SSH two thirds of reports were in English. English-language use also varied by reviewer characteristics. It was lower among female reviewers overall, mainly because more female reviewers were in SSH. It was also lower for SSH reviewers from Germany and France than for reviewers from English-speaking countries. Over time, the proportion of English-language reviews increased to over 90%, mainly because English use rose in SSH.

We expanded our comment in the Discussion and mention the new table:

In addition, because non-English reports were excluded, our findings apply only to English-language peer review reports. This includes virtually all reports in LS and MINT, but only about two-thirds of reports in SSH (see S3 Table). In SSH, the proportion of English-language reports increased from around 55% in 2016 to around 70% or more from 2020 onward, suggesting that any bias introduced by excluding non-English reports was greater in the earlier part of the study period. Nevertheless, the results should be interpreted with caution for SSH, where inclusion of non-English reports might have changed the observed patterns.

S3 Table: *The prevalence of English-language reviews by reviewer characteristics.* Each cell shows the count of all reviews (English and other languages combined) and the percentage of English-language reviews within that category.

	LS	MINT	SSH	Total
**Reviews**	**15,162 (99.6%)**	**15,680 (98.9%)**	**12,964 (68.4%)**	**43,806 (90.1%)**
**Gender of Reviewer**				
Female	3,507 (99.7%)	1,915 (99.2%)	5,164 (66.1%)	10,586 (83.2%)
Male	11,655 (99.5%)	13,765 (98.8%)	7,800 (69.9%)	33,220 (92.3%)
**Country of Affiliation**				
United States of America	3,813 (100.0%)	3,391 (99.9%)	2,083 (99.3%)	9,287 (99.8%)
Germany	1,220 (98.1%)	1,520 (95.4%)	2,310 (35.2%)	5,050 (68.5%)
Great Britain and N. Ireland	1,078 (100.0%)	1,183 (100.0%)	1,084 (99.4%)	3,345 (99.8%)
France	732 (99.0%)	908 (95.7%)	857 (28.8%)	2,497 (73.7%)
Italy	535 (100.0%)	939 (100.0%)	340 (83.2%)	1,814 (96.9%)
Canada	517 (100.0%)	586 (99.5%)	487 (70.4%)	1,590 (90.8%)
Netherlands	585 (100.0%)	368 (99.7%)	428 (98.1%)	1,381 (99.3%)
Australia	527 (100.0%)	388 (100.0%)	278 (100.0%)	1,193 (100.0%)
Other	2,759 (99.9%)	3,749 (99.9%)	1,670 (81.3%)	8,178 (96.1%)
Unknown	3,396 (99.1%)	2,648 (97.8%)	3,427 (57.8%)	9,471 (83.8%)
**Submission Year**				
2016	881 (99.7%)	917 (98.5%)	933 (56.9%)	2,731 (84.7%)
2017	1,808 (99.5%)	1,897 (98.2%)	1,559 (62.0%)	5,264 (87.9%)
2018	1,982 (99.4%)	2,052 (98.5%)	1,664 (65.3%)	5,698 (89.1%)
2019	1,948 (99.3%)	2,020 (98.8%)	1,815 (68.8%)	5,783 (89.5%)
2020	2,264 (99.5%)	2,510 (99.0%)	2,129 (71.3%)	6,903 (90.7%)
2021	2,444 (99.6%)	2,474 (99.2%)	2,072 (73.5%)	6,990 (91.7%)
2022	2,520 (99.8%)	2,543 (99.2%)	1,801 (72.6%)	6,864 (92.4%)
2023	1,315 (99.8%)	1,267 (99.2%)	991 (69.4%)	3,573 (91.2%)
